# Differentiation and Characterization of Excitatory and Inhibitory Synapses by Cryo-electron Tomography and Correlative Microscopy

**DOI:** 10.1523/JNEUROSCI.1548-17.2017

**Published:** 2018-02-07

**Authors:** Chang-Lu Tao, Yun-Tao Liu, Rong Sun, Bin Zhang, Lei Qi, Sakar Shivakoti, Chong-Li Tian, Peijun Zhang, Pak-Ming Lau, Z. Hong Zhou, Guo-Qiang Bi

**Affiliations:** ^1^National Laboratory for Physical Sciences at the Microscale,; ^2^Chinese Academy of Sciences Key Laboratory of Brain Function and Disease,; ^3^School of Life Sciences,; ^4^Chinese Academy of Sciences Center for Excellence in Brain Science and Intelligence Technology, Innovation Center for Cell Signaling Network, University of Science and Technology of China, Hefei, Anhui 230026, China,; ^5^Division of Structural Biology, Wellcome Trust Centre for Human Genetics, University of Oxford, Oxford OX37BN, United Kingdom,; ^6^The California NanoSystems Institute, and; ^7^Department of Microbiology, Immunology and Molecular Genetics, University of California, Los Angeles, Los Angeles, California 90095

**Keywords:** correlative light and electron microscopy, cryo-electron tomography, neurotransmitter receptor, postsynaptic density, synaptic ultrastructure, synaptic vesicle

## Abstract

As key functional units in neural circuits, different types of neuronal synapses play distinct roles in brain information processing, learning, and memory. Synaptic abnormalities are believed to underlie various neurological and psychiatric disorders. Here, by combining cryo-electron tomography and cryo-correlative light and electron microscopy, we distinguished intact excitatory and inhibitory synapses of cultured hippocampal neurons, and visualized the *in situ* 3D organization of synaptic organelles and macromolecules in their native state. Quantitative analyses of >100 synaptic tomograms reveal that excitatory synapses contain a mesh-like postsynaptic density (PSD) with thickness ranging from 20 to 50 nm. In contrast, the PSD in inhibitory synapses assumes a thin sheet-like structure ∼12 nm from the postsynaptic membrane. On the presynaptic side, spherical synaptic vesicles (SVs) of 25–60 nm diameter and discus-shaped ellipsoidal SVs of various sizes coexist in both synaptic types, with more ellipsoidal ones in inhibitory synapses. High-resolution tomograms obtained using a Volta phase plate and electron filtering and counting reveal glutamate receptor-like and GABA_A_ receptor-like structures that interact with putative scaffolding and adhesion molecules, reflecting details of receptor anchoring and PSD organization. These results provide an updated view of the ultrastructure of excitatory and inhibitory synapses, and demonstrate the potential of our approach to gain insight into the organizational principles of cellular architecture underlying distinct synaptic functions.

**SIGNIFICANCE STATEMENT** To understand functional properties of neuronal synapses, it is desirable to analyze their structure at molecular resolution. We have developed an integrative approach combining cryo-electron tomography and correlative fluorescence microscopy to visualize 3D ultrastructural features of intact excitatory and inhibitory synapses in their native state. Our approach shows that inhibitory synapses contain uniform thin sheet-like postsynaptic densities (PSDs), while excitatory synapses contain previously known mesh-like PSDs. We discovered “discus-shaped” ellipsoidal synaptic vesicles, and their distributions along with regular spherical vesicles in synaptic types are characterized. High-resolution tomograms further allowed identification of putative neurotransmitter receptors and their heterogeneous interaction with synaptic scaffolding proteins. The specificity and resolution of our approach enables precise *in situ* analysis of ultrastructural organization underlying distinct synaptic functions.

## Introduction

Chemical synapses are basic functional units in neural circuits for information transmission, processing, and storage (Eccles, 1964; Südhof and Malenka, 2008; Mayford et al., 2012). The brain's remarkable computational power and cognitive capacity stem from the enormous number of synapses in the brain, the plasticity of each synapse, and the molecular and functional diversity across these synapses (Milner et al., 1998; Bi and Poo, 2001). Glutamatergic and GABAergic synapses, the two main types of central synapses, play opposite roles in excitation and inhibition. They have been shown by biochemical and electrophysiological studies to contain different sets of molecular and cellular components and to exhibit distinct functional properties and plasticity rules (Craig and Boudin, 2001; Südhof and Malenka, 2008; Vogels and Abbott, 2009; Sassoè-Pognettoet al., 2011). How are these different components organized into the intricate machinery to perform distinct synaptic functions? Electron microscopy (EM) has been a primary tool for addressing this question by enabling the visualization of the ultrastructure of various synapses (Siksou et al., 2007; Harris and Weinberg, 2012).

Classical EM uses chemical fixation, dehydration, and plastic embedding, followed by sectioning and heavy-metal staining to image brain tissues and cultured neurons. Such meticulous processing has enabled the use of electron beams to image various biological specimens at high contrast. Indeed, classical EM observations have shaped much of our current knowledge about synaptic ultrastructure (Sorra and Harris, 2000; Harris and Weinberg, 2012). For example, prominent subcellular features, such as the postsynaptic density (PSD) and synaptic vesicles (SVs), are well documented, especially for excitatory synapses (Gray, 1959; Colonnier, 1968; Schikorski and Stevens, 1997; Harris and Weinberg, 2012). The 3D resolving capability of electron tomography (ET) has yielded better views of synaptic ultrastructure (Harlow et al., 1998; Ress et al., 2004; Burette et al., 2012). The improved structural preservation provided by high-pressure freezing with freeze substitution (HPF-FS; Tatsuoka and Reese, 1989) combined with ET (Rostaing et al., 2006) has allowed studies of the organization and dynamics of SVs (Siksou et al., 2007; Watanabe et al., 2013; Imig et al., 2014; Jung et al., 2016) and the 3D organization of macromolecular complexes in individual synapses, such as the PSD-95/glutamate receptor complex at the PSD (Chen et al., 2008, 2015).

It remains challenging to characterize the structure and organization of cellular and molecular machinery of specific synaptic types at higher resolution (Hurbain and Sachse, 2011; Tao et al., 2012), because damage or deformation from sample preparation procedures can complicate structural interpretation (Hurbain and Sachse, 2011). Cryo-electron tomography (cryo-ET), which aims to overcome this limitation, has been used to visualize distribution of SVs and other ultrastructural features in isolated synaptosomes and cryo-sections of brain tissues (Fernández-Busnadiegoet al., 2010; Shi et al., 2014; Wilhelm et al., 2014; Perez de Arce et al., 2015). However, cryo-ET alone cannot unambiguously identify synapse types due to a lack of specific labeling, such as immunogold staining or photoconversion of diaminobenzidine for classical EM (Megías et al., 2001; Schikorski and Stevens, 2001; Rostaing et al., 2006; Harris and Weinberg, 2012). One way to overcome this shortcoming is to take advantage of the molecular specificity of fluorescence labeling and sample preservation of cryo-ET in cryo-correlative light microscopy (LM) and EM (cryo-CLEM), as suggested previously (Lucić et al., 2007), although the ability of this approach to distinguish different synapse types is yet to be realized. In the current study, we developed an efficient cryo-CLEM platform to identify different types of synapses in cultured hippocampal neurons, and to define presynaptic and postsynaptic ultrastructural features of excitatory and inhibitory synapses in their native state. By high-resolution cryo-ET with cutting-edge direct electron detection (Li et al., 2013), Volta phase plate (VPP; Danev et al., 2014; Fukuda et al., 2015), and electron energy filter (Verbeeck et al., 2004) technologies, we could also visualize putative glutamate receptors and GABA_A_ receptors (GABA_A_Rs) and their organization at the postsynaptic membrane of excitatory and inhibitory synapses.

## Materials and Methods

**Figure 1.**
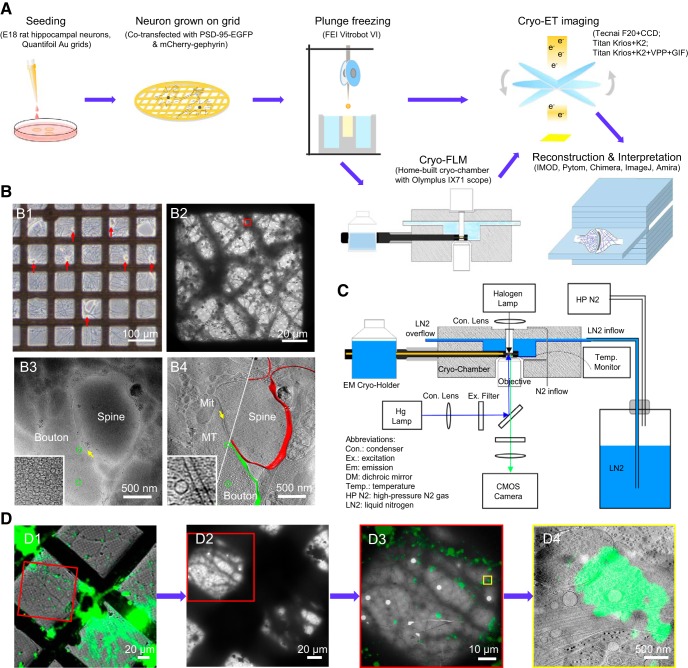
Imaging primary rat hippocampal neurons with cryo-ET/cryo-CLEM. ***A***, Illustration of the workflow of cryo-ET/cryo-CLEM imaging of neurons grown on gold EM grids. ***B***, Representative results from different stages of the workflow. ***B1***, LM image of cultured neurons (red arrows indicate cell bodies). ***B2***, Cryo-EM image of neuronal processes in one grid square. ***B3***, A single cryo-EM projection image of the boxed area in ***B2*** showing a synapse-like structure with a presynaptic bouton (Bouton) containing a dense population of SVs (green circles), a postsynaptic spine (Spine), and a relatively uniform cleft (yellow arrow). Inset shows a zoomed-in view of the synaptic bouton area with a dense population of SVs. ***B4***, A tomographic slice showing fine structure of the same synapse in ***B3***, which was identified as a spine synapse by following through the tomogram in 3D, with mitochondrion (Mit), microtubules (MT), and SVs (green circles) and superposed with segmented presynaptic membrane (green) and postsynaptic membrane (red). Inset shows a zoomed-in view of the synaptic cleft area with transcleft structures. ***C***, Schematics depicting main components of cryo-fluorescence light microscope with an EM cryo-holder. ***D***, Pipeline of imaging synapse with cryo-CLEM. ***D1***, Merged cryo-fluorescence and cryo-bright-field light images. ***D2***, Low-magnification cryo-EM image including the same grid square. ***D3***, Merged images of boxed area in ***D1*** and ***D2*** after fine alignment. ***D4***, Tomographic slice of the boxed area in ***D3*** superimposed with aligned fluorescence image showing the structure of a synapse with a green fluorescent punctum.

The overall workflow of experimental procedures is illustrated in Figure 1*A*. Primary neuronal cultures were grown on EM grids and then plunge-frozen for cryo-ET imaging followed by 3D reconstruction. For some cultures transfected with constructs of fluorescent protein-tagged synaptic proteins, cryo-fluorescence microscopy was performed before cryo-ET for correlative imaging. All animal procedures were performed following the guidelines of the Animal Experiments Committee at the University of Science and Technology of China.

### 

#### 

##### Primary culture of hippocampal neurons.

Low-density cultures of dissociated embryonic rat hippocampal neurons were prepared as previously described (Bi and Poo, 1998) with modifications. Quantifoil R2/2 gold EM grids (200 mesh with holey carbon film of 2 μm hole size and 2 μm spacing) or Quantifoil R2/2 gold NH2 finder grids (100 mesh with holey carbon film of 2 μm hole size and 2 μm spacing) were plasma-cleaned with H_2_ and O_2_ for 10 s using a plasma cleaning system (Gatan), and sterilized with UV light for 30 min. These grids were then coated with poly-l-lysine (Sigma-Aldrich) overnight, followed by washing with HBSS and double-distilled H_2_O for ∼12 h each. Hippocampi were removed from embryonic day 18 rats (without distinguishing sex differences) and were treated with trypsin for 15 min at 37°C, followed by washing and gentle trituration. The dissociated cells were plated on the poly-l-lysine-coated EM grids in 35 mm Petri dishes at a density of 40,000–60,000 cells/ml, and maintained in incubators at 37°C in 5% CO_2_. The culture medium was NeuroBasal (Invitrogen) supplemented with 5% heat-inactivated bovine calf serum (PAA Laboratories) plus 5% heat-inactivated fetal bovine serum (HyClone), 1× Glutamax (Invitrogen), and 1× B27 (Invitrogen). Twenty-four hours after plating, half of the medium was replaced by serum-free culture medium. Subsequently, one-third of the culture medium was replaced with fresh culture medium twice a week. For correlative microscopy, cultures were coinfected with lentiviruses encoding PSD-95-EGFP and mCherry-gephyrin constructs (see below) for 5–7 d *in vitro* (DIV) before vitrification of the grid. Twelve hours after the infection, half of the culture medium was replaced by fresh medium.

To prevent overgrowth of glial cells, the cultures were treated with cytosine arabinoside (Sigma-Aldrich) at various stages. Cultures were used for cryo-EM imaging at 14–18 DIV, when healthy, low-density cultures formed patches of monolayer neuronal processes (Fig. 1*B1*). We judge whether the culture is healthy based on morphological criteria, e.g., smooth soma and dendrites with multiple branches viewed under phase-contrast LM, and ≥1 probable synapse each few micrometers along the dendrites of transfected neurons, as viewed under fluorescence microscopy. According to our experience, such criteria predict retention of functional properties of synaptic transmission and plasticity evaluated with patch-clamp recording and calcium imaging.

##### DNA constructs and lentivirus preparation.

The PSD-95 cDNA was amplified from GW-PSD-95-EGFP plasmid (a generous gift from Dr. Weidong Yao) and subcloned into pLenti-CaMKII-mKate2 vector to produce pLenti-CaMKII-PSD-95-mKate2. The EGFP cDNA was amplified from the pEGFPN1 plasmid, and then subcloned into pLenti-CaMKII-PSD-95-mKate2 to produce the pLenti-CaMKII-PSD-95-EGFP plasmid. The mCherry-gephyrin lentiviral construct (Dobie and Craig, 2011) was a generous gift from Dr. Ann Marie Craig. Both PSD-95-EGFP and mCherry-gephyrin lentiviral constructs were packaged into lentivirus following a protocol from Dr. Karl Deisseroth's laboratory (Zhang F et al., 2010).

##### Frozen-hydrated sample preparation.

After being removed from the CO_2_ incubator, low-density neuronal cultures (14–18 DIV) on EM grids were first placed in extracellular solution (ECS; containing 150 mm NaCl, 3 mm KCl, 3 mm CaCl_2_, 2 mm MgCl_2_, 10 mm HEPES, and 5 mm glucose, pH 7.3), then mounted on a Vitrobot IV (FEI). Protein A-coated colloidal gold beads (15 nm; CMC) were added to the grid (4 μl each, stock solution washed in ECS and diluted 10 times after centrifugation) as fiducial markers. The grids were then plunged into liquid ethane for rapid vitrification of the samples, which were then stored in liquid nitrogen until use.

##### Cryo-ET imaging.

Cryo-ET data were collected with single-axis tilt using either a Tecnai F20 transmission electron microscope (FEI) equipped with an Eagle 4K × 4K multiport CCD camera (FEI), or a Titan Krios (FEI) with a K2 Summit direct electron detector (K2 camera, Gatan). The Tecnai F20 was operated at an acceleration voltage of 200 kV. Tilt series were collected from −60 to +60° at of 2° intervals using FEI Xplore 3D software, with the defocus value set at −12 to −18 μm, and the total electron dosage of ∼100 e^−^/Å^2^. The final pixel size was 0.755 nm. The Titan Krios was operated at an acceleration voltage of 300 kV, with or without VPP and Gatan image filter (GIF). In either configuration, images were collected by the K2 camera in counting mode. In the absence of VPP and GIF, tilt series were acquired from −64 to +64° at 2° intervals using Leginon (Suloway et al., 2005), with the defocus value maintained at −10 μm, and the total accumulated dose of ∼120 e^−^/Å^2^. The final pixel size was 0.765 nm. When VPP and GIF were used, the energy filter slit was set at 20 eV, and VPP was conditioned by preirradiation for 60 s to achieve an initial phase shift of ∼0.3π (Fukuda et al., 2015). Tilt series were acquired from −66 to +60° at an interval of 2 or 3° using SerialEM (Mastronarde, 2005) with the defocus value maintained at −1 μm and the total accumulated dose of ∼150 e^−^/Å^2^. The final pixel size was 0.435 nm.

For this study, we examined 78 grids, of which 12 were used for data collection. The rest were discarded because the grids were damaged during transfer or freezing, cultures were too dense and/or too thick, or cultures appeared not healthy with few or no synapses found. Usually 3–5 grid squares (each ∼100 × 100 μm^2^) per grid were selected for imaging. To obtain high-quality cryo-ET images, it is critical to choose thin culture areas with healthy yet relatively low-density dendrites (Fig. 1*B2*). Generally, areas >∼500 nm thick were ignored. At this thickness, subcellular structures, such as mitochondria, microtubules, and SVs, could not be distinguished in single-projection images.

##### Cryo-correlative light and electron microscopy imaging.

The hardware of our cryo-light microscope system includes a custom-built cryo-chamber with liquid nitrogen supply, a Gatan 626 EM cryo-holder, and an Olympus IX71 inverted fluorescence microscope (Fig. 1*C*). The inside channel of the cryo-chamber was precooled to −190°C by liquid nitrogen, and maintained below −180°C, as monitored by a thermoelectric sensor. Nitrogen gas flowed through the objective lens and light-source windows during the experiment to prevent frost accumulation. Then, an EM grid with frozen-hydrated sample was loaded onto an EM cryo-holder, which was subsequently inserted into the cryo-chamber.

For cryo-CLEM imaging, fluorescence images were taken using a 40× air-objective lens (Olympus LUCPLFLN 40×; numerical aperture, 0.6) and an ANDOR NEO sCMOS camera (Andor) attached to the fluorescence microscope. For each field of view, three images were collected, one in bright field, another in the EGFP channel [exciter (Ex): 470/40; dichroic mirror (DM): 495; emitter (Em): 525/50; Chroma, 49002], and the third in the mCherry channel (Ex: 562/40; DM: 593; Em: 641/75; Semrock, mCherry-B-000). Typically, ∼10 sets of images were sufficient to cover all good areas (∼40 squares) on each grid, which took ∼20 min to complete; a “good area” was defined as a grid square (∼100 × 100 μm^2^) of appropriate sample thickness that displayed multiple dendritic branches, usually containing dozens of PSD-95-EGFP puncta or multiple mCherry-gephyrin puncta under fluorescence microscopy (Fig. 1*D1*).

Immediately after the LM imaging, the EM cryo-holder with grid was directly transferred into a Tecnai F20 scope. Areas of the sample imaged in cryo-light microscope were identified in the EM using the indexes of the finder grids (Fig. 1*D1*,*D2*). Low-magnification (330×) EM images were collected and approximately aligned with bright-field LM images using Midas program in the IMOD package (RRID:SCR_003297; Kremer et al., 1996). After rough alignment, a set of holes on the carbon layer of the grid were picked from both the low-magnification EM images and their corresponding fluorescence images using 3dmod in the IMOD package. Transformation functions between the EM and LM images were calculated based on the selected positions by minimizing the mean squared error.

When the low-magnification EM image and LM image were optimally aligned (Fig. 1*D3*), ∼15 holes on carbon (in one square) in the low-magnification EM image were selected, with their pixel positions recorded. The same holes were identified at 5000× magnification and the mechanical coordinates were recorded. Afterward, the transformation function from the pixel positions to EM mechanical coordinates was determined using similar linear regression methods. With the transformation functions, positions of selected fluorescent puncta (putative excitatory or inhibitory synapses) were converted into corresponding EM mechanical coordinates, which were used to guide EM-image acquisition. Tilt series were collected on the area with selected fluorescent signals. Finally, tomographic slices were fine-aligned and merged with the fluorescence images to identify each synapse (Fig. 1*D4*) using Midas and ImageJ (RRID:SCR_003070). Python scripts (RRID:SCR_008394) to integrate the correlation procedures are available to interested readers upon request.

##### Three-dimensional reconstruction and rendering.

Tilt series were aligned and reconstructed using IMOD. Gold beads added to the sample before plunge freezing were used as fiducial markers to align the tilt series. Reconstruction was performed using a simultaneous iterative reconstruction technique with 5 or 15 iterations. A fraction of the data collected was discarded during reconstruction for technical reasons: for Tecnai F20, ∼50% of the data were discarded because of such issues as stage drift, beam blockade at high tilt angles, and occasional autofocus failure; for Titan Krios, <25% of the data were discarded, usually because of issues with beam blockade or autofocus failure at high tilt angle, GIF mistuning, or errors in VPP charging.

Cellular structures, including membranes, actin filaments, microtubules, mitochondria, endoplasmic reticulum, and putative membrane proteins in the tomograms were segmented by manually selecting areas containing corresponding structures in UCSF Chimera (RRID:SCR_004097; Pettersen et al., 2004) and filtered to make the densities smooth and continuous. The volume of each structure was displayed according to the intensity value. The human 80S ribosome structure (Electron Microscopy Data Bank accession code EMD-5224; Brandt et al., 2010), after low-pass filtering and scaling to the same pixel size as the tomograms, was used as the template to localize ribosomes in the synapses using PyTom (Hrabe et al., 2012). The template structures were then placed in the final segmentation using UCSF Chimera. SVs were identified as described below and rendered based on their size. The receptor-like structures and their interactions with other structures on the cytoplasmic or the cleft side (see Figs. 8*G*, 9*G*) were manually segmented in the Amira software package (RRID:SCR_014305).

##### Quantitative analyses of PSD and synaptic cleft.

To analyze PSD profiles, we extracted a 10-nm-thick (*z*) subvolume containing a PSD from each synapse (the *z*-axis is parallel with the direction of the electron beam; *y*-axis is along the tilt axis; and *x*-axis is the direction perpendicular to the *xz* plane). Virtual slices within the slab were averaged along the *z*-axis using the Slicer tool in 3dmod to create a 2D projection (*x*, *y*). Then, a contour line was manually drawn to trace the postsynaptic membrane in this projection using 3dmod, and a set of virtual lines parallel to this contour line were defined at different distances from the contour line. Averaging along each virtual line yields a cross-sectional mean density value; the mean density values corresponding to different distances from the postsynaptic membrane constitute the density profile for the synapse. To compensate for variability in imaging conditions, the density profile was normalized by subtracting the mean value corresponding to a “flat” region 100 to 200 nm from the postsynaptic membrane, and then dividing by the SD of this “flat” region.

The density profile typically consists of distinct peaks corresponding to the presynaptic and postsynaptic membranes as well as the part of PSD with highest density (hereafter referred to as the PSD peak); the positions of these components were measured by Gaussian fitting around the corresponding peaks in the profile (see Fig. 4*A*2,*B2*). Synaptic cleft width was defined as the distance from the center of the postsynaptic membrane to the center of the presynaptic membrane. The position of the PSD peak (i.e., its distance from the postsynaptic membrane) was defined as *d1*. From the PSD peak to the flat background into the postsynaptic side, the density profiles varied widely. A simple, objective approach to quantify this was to fit it with an exponential decay function, as follows: *v* = *Ae*^−*d*/λ^ + *B*, where *d* is the distance to PSD peak, ν is density value, and λ is the length constant of the fitted curve. Combined with the distance of the PSD peak from the postsynaptic membrane, we define *d2* = *d1* + λ, and consider *d2* as a measure of the thickness of the PSD.

##### Quantitative analysis of SVs.

Size range and shape variation of all SVs were initially analyzed in two steps. First, we used template matching to identify vesicles. To do this, a set of featureless spherical shells 5 nm thick with diameter ranging from 25 to 70 nm at 1 nm intervals were designed as templates. These templates were Gaussian low-pass filtered to 10 nm resolution with EMAN2.1, and used for template matching of SVs in the tomograms using PyTom. The results of template matching were evaluated by visual inspection, and mismatches were discarded. Second, we performed shape analysis by 2D fitting. For each template-matched vesicle, the central tomographic slice was extracted and the vesicular membrane was masked using a donut-shaped mask. Then the coordinates of the pixels in the masked vesicle, whose density value was higher than the average pixel density of the masked vesicle, were used for 2D elliptic fitting (least-square solution of the ellipse's implicit polynomial representation in Python implementation; Fitzgibbon et al., 1999). A few vesicles (mostly ellipsoidal) could not be detected using template matching. For those vesicles, we manually picked points on the SV membrane on the central slice of those vesicles using the contour tool in 3dmod, and used those points for 2D elliptic fitting.

For 3D fitting analysis of selected vesicles, we first manually picked ≥18 points on the membrane of each vesicles in 3D using the contour tool in 3dmod. These picked points were fitted to an ellipsoid using least-square solution of the ellipsoid's implicit polynomial representation in a Matlab implementation (RRID:SCR_001622). The lengths of all axes in each vesicle were recorded. Note that the tomograms had nonisotropic resolution (the resolution along *z* direction of the reconstructed 3D tomogram is much lower than the *x*/*y* resolution) because of missing-wedge effect due to the limited range of tilt angles. This causes the EM density along *z* direction to be blurred, but does not bias the shape of fitted vesicles.

##### Analysis of receptor-like structures.

To analyze receptor-like structures, two high-quality synaptic tomograms, which were collected using a Titan Krios equipped with K2 camera, VPP, and GIF, were selected. One of them contained an excitatory spine synapse and the other an inhibitory shaft synapse. We extracted subvolumes (*x*, *y*, *z*) containing particles on the cleft side of the postsynaptic membrane of the excitatory and the inhibitory synapse and performed the following analyses. Each subvolume was averaged (9.6 and 6.5 nm for the excitatory and inhibitory synapse, respectively) along the *z*-axis (parallel to the direction of the electron beam) to create a 2D projection (*x*, *y*; multiple virtual slices averaging using the Slicer tool in 3dmod). Particles with shape and size similar to those of known receptor structures were visually classified as putative receptors, and the rest were classified as putative nonreceptor structures. The length and width of each particle were also measured manually in a blind manner (independent of the visual classification). After that, projections of all particles classified as glutamate receptors or GABA_A_Rs were aligned by matching the postsynaptic membrane-end of all these particles, and rotating the long axis of the particles to the vertical direction. Aligned projections were averaged subsequently using the Slicer tool in 3dmod.

To obtain the sizes (length and width) of specific transmitter receptors based on the known crystal structures, density maps of AMPA receptors (AMPARs), NMDA receptors (NMDARs), and GABA_A_Rs were stimulated and low-pass filtered to 27 Å resolution using e2pdb2mrc.py program in EMAN2.1 from their atomic models [NMDAR, PDB: 4TLL (Lee et al., 2014); AMPAR, PDB: 4U2P (Dürr et al., 2014); GABA_A_R, PDB: 4COF (Miller and Aricescu, 2014)], respectively. The low-pass-filtered density maps were projected using the Slicer tool in 3dmod with 9° spacing of the projection angles perpendicular to the longest axis of the receptor density, generating 20 projections from each density map. We measured the sizes of each projection to get mean length and width with SDs for each receptor's extracellular domain.

##### Experimental design and statistical analysis.

**Figure 2.**
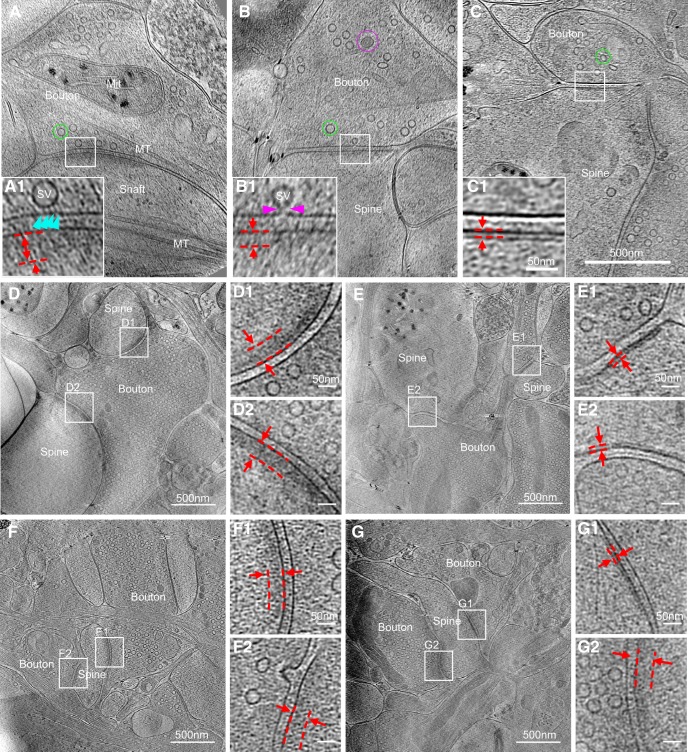
Synapses of various sizes, shapes, and ultrastructural details imaged with cryo-ET. ***A–C***, Three tomographic slices showing structures of different synapses. In the synapses, structures, such as SVs and dense core vesicle in presynaptic boutons (Bouton), microtubules (MT) in boutons and dendritic shaft (Shaft), mitochondria (Mit) in presynaptic bouton and postsynaptic spine (Spine), are clearly visible. ***A1–C1***, Zoomed-in views of corresponding boxed areas from ***A–C*** showing thick (***A1***, ***B1***, dashed parallel lines) and thin (***C1***, dashed parallel lines) PSDs, as well as SVs attached (***A1***, cyan arrowhead) or fused (***B1***, pink arrowheads) to the presynaptic membrane. ***D***, ***E***, Two synapses sharing the same presynaptic axon (determined by following through their tomograms in 3D), both with thick PSDs (***D1*** and ***D2***) or both with thin PSDs (***E1*** and ***E2***), respectively. ***F***, ***G***, Two synapses sharing the same postsynaptic spine, both with thick PSDs (***F1*** and ***F2***), or one with thin PSD (***G1***) and the other with thick PSD (***G2***).

**Figure 3.**
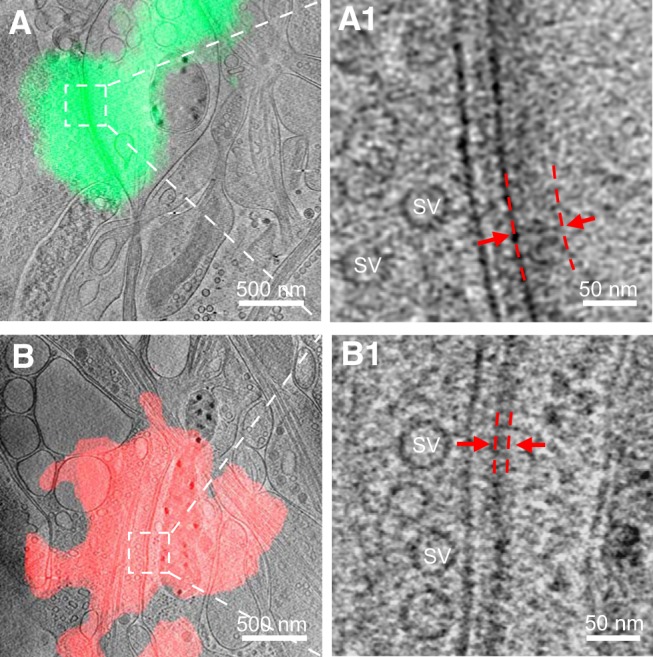
Identification of excitatory and inhibitory synapses with cryo-CLEM. ***A***, ***B***, Tomographic slices of an excitatory (***A***) and inhibitory (***B***) synapse colocalized with PSD-95-EGFP and mCherry-gephyrin puncta, respectively. ***A1***, ***B1***, Zoomed-in views of the boxed area in ***A*** and ***B*** showing the synapse with thick and thin PSD, respectively. Red dashed lines indicate the range of the PSD.

**Figure 4.**
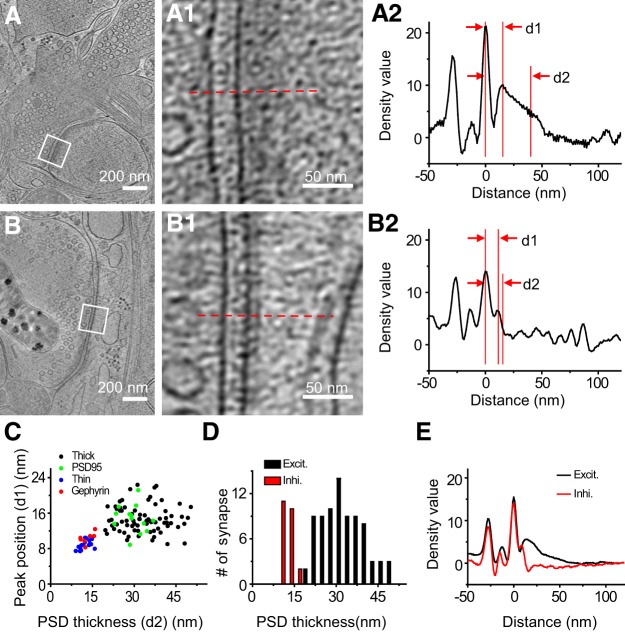
Quantitative and statistical characterization of excitatory and inhibitory PSDs. ***A***, ***B***, Tomographic slices of two synapses with thick and thin PSD respectively. ***A1***, ***B1***, Zoomed-in views of the marked areas in ***A*** and ***B***. ***A2***, ***B2***, Normalized density profiles of the two synapses in ***A*** and ***B*** respectively with cross-sectional mean density plotted against distance to postsynaptic membrane (see Materials and Methods). On the *x*-axis of this plot, 0 was set to be the position of the postsynaptic membrane, and positive values are on the postsynaptic side. The density profiles were normalized against the density values at distances ranging from 100 to 200 nm such that the average density value in this range is zero and their SD is unity. *d1*, PSD peak position; *d2* is the sum of *d1* and the length constant obtained from the exponential fit of the profile from *d1* to the flattened background, to provide a measure of the thickness of the PSD (see Materials and Methods). ***C***, Scatter plot of PSD peak position and PSD thickness of all synapses show two well defined clusters. ***D***, Histogram shows the PSD thickness distribution of all excitatory and inhibitory synapses respectively. ***E***, Averaged density curve of all excitatory synapses and all inhibitory synapses respectively.

**Figure 5.**
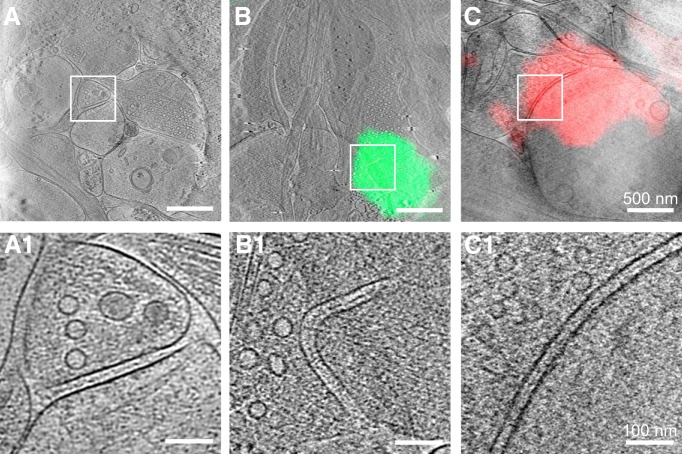
Synapses without visible PSDs. ***A***, A 15-nm-thick tomographic slice of an unidentified synapse imaged by cryo-ET only. ***B***, ***C***, A 15-nm-thick tomographic slice of an excitatory (***B***) and an inhibitory (***C***) synapse identified by cryo-CLEM superposed with the fluorescence image of colocalized PSD-95-EGFP and mCherry-gephyrin, respectively. ***A1–C1***, Zoomed-in views of the boxed area in ***A–C*** respectively showing that no PSD structures are visible in these synapses.

**Figure 6.**
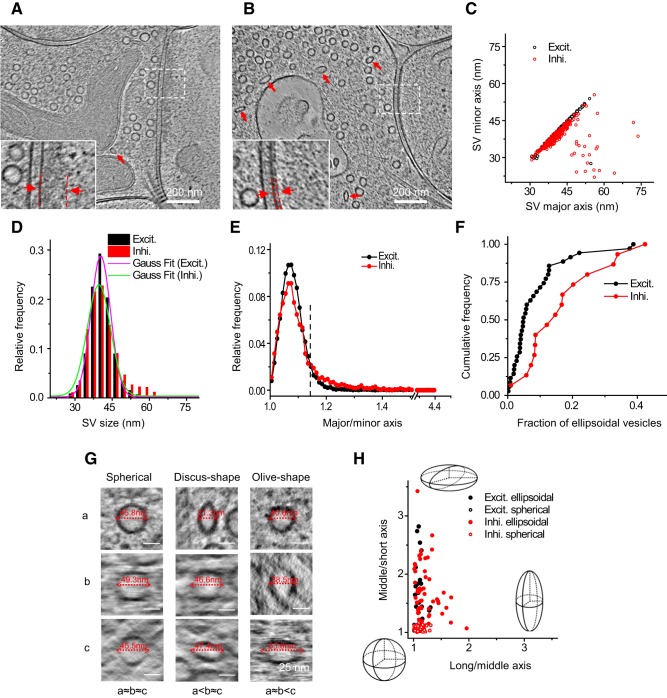
Heterogeneity of synaptic vesicles in excitatory and inhibitory synapses. ***A***, ***B***, Tomographic slices of an excitatory and an inhibitory synapse respectively. Insets are zoomed-in views showing thick and thin PSDs from ***A*** and ***B*** respectively. ***C***, Scatter plot showing the major and minor axes of SVs in the two synapses in ***A*** and ***B*** measured by 2D fitting. ***D***, Distribution of vesicle sizes in excitatory and inhibitory synapses (16,476 vesicles in 35 excitatory synapses and 4766 vesicles in 15 inhibitory synapses). ***E***, Distribution of ellipticity of SVs (major to minor axis ratio) in excitatory and inhibitory synapses. A threshold (dashed line) was set at major/minor = 1.14, which is approximately twice the peak position (major/minor = 1.07) from perfect circle (major/minor = 1) to separate ellipsoidal from spherical vesicles. Coincidentally, this threshold is also close to the cross point of the two distribution curves. ***F***, Cumulative frequency of the fraction of ellipsoidal vesicles in excitatory and inhibitory synapses. ***G***, Tomographic slices of spherical (left column), discus-shaped (i.e., oblate spheroid, middle column), and olive-shaped (i.e., prolate spheroid, right column) SVs viewed in three orthogonal planes rotated so that each of the three principle axes (a–c) of the vesicles can be measured horizontally in the corresponding plane. For the spherical vesicles, a≈b≈c; for discus-shaped ones, a<b≈c; for olive-shaped ones, a≈b<c. ***H***, Long to middle axis ratio versus middle to short axis ratio of 70 ellipsoidal SVs and 70 adjacent spherical SVs in five excitatory and five inhibitory synapses. Schemes depict the shapes at the given positions in the plot.

**Figure 7.**
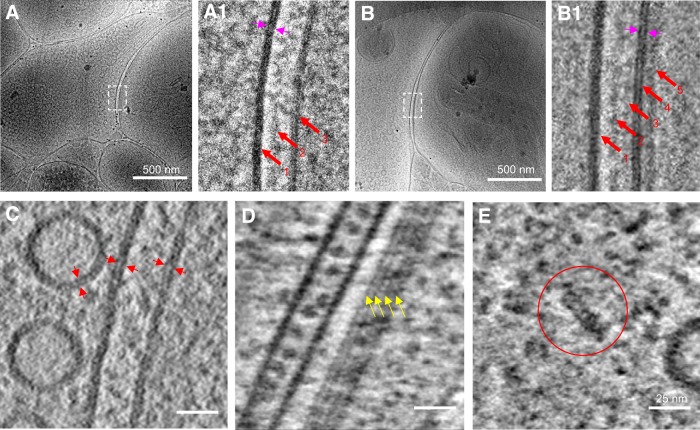
High-resolution cryo-ET of synapses obtained using VPP, electron energy filter, and direct electron detection. ***A***, ***B***, Single-projection cryo-EM images of an excitatory and inhibitory synapse respectively. ***A1***, Zoomed-in view of the white boxed area in ***A*** showing three bands of increased density (red arrows) at the junctional area: presynaptic membrane, intercleft band, and postsynaptic membrane. The two leaflets of membrane bilayer can be distinguished (paired magenta arrows). ***B1***, Zoomed-in view of the white boxed area in ***B*** showing five density bands (red arrows) at the junction: presynaptic membrane, two intercleft bands, postsynaptic membrane, and postsynaptic density. Two leaflets of membrane bilayer can also be distinguished (paired magenta arrows). ***C***, Two leaflets of membrane bilayer (paired arrowheads) at SVs and synaptic membrane were evident in the reconstructed tomographic slice. ***D***, ***E***, Tomographic slices showing macromolecular structures, such as microtubule protofilaments (***D***, yellow arrows) and proteasome-like particle (***E***, red circle) in different synapses.

**Figure 8.**
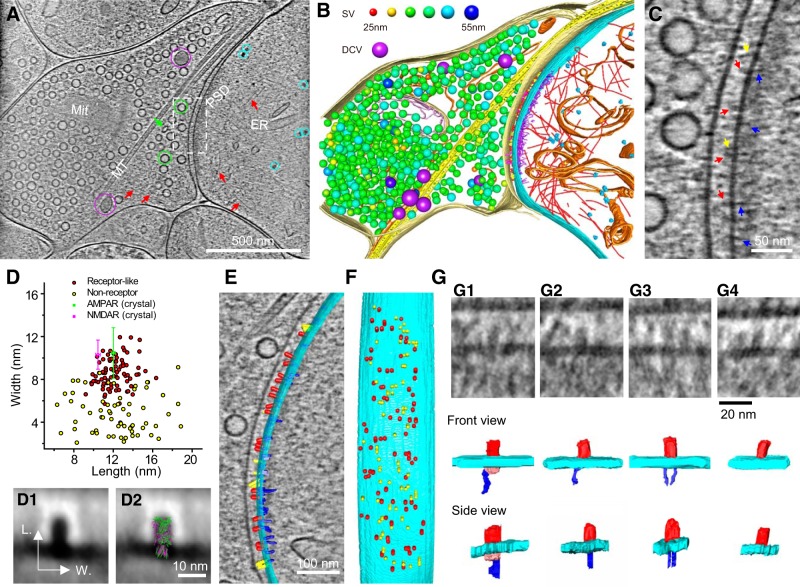
Putative receptors and scaffolding proteins in an excitatory synapse. ***A***, An 8.7-nm-thick tomographic slice of an excitatory synapse. Circles: SVs (green), DCVs (purple), ribosome-like structures (cyan); arrows: ellipsoidal vesicle (green), putative actin filaments (red). ER, Endoplasmic reticulum; Mit, mitochondria; MT, microtubule. ***B***, 3D segmented structures of the whole tomogram (∼300 nm thickness) of the same synapse shown in ***A*** rendered as surfaces, colored as follows: outer-Mit, gold; inner-Mit, light pink; MT, yellow; ER, orange; ribosomes, cyan; actin filaments, red; presynaptic membrane, light yellow; postsynaptic membrane, cyan; presynaptic putative adhesion molecules, magenta; postsynaptic putative adhesion molecules, yellow; putative glutamate receptors, red; PSD filaments attached to the postsynaptic membrane, blue; PSD filaments away from the postsynaptic membrane, purple. Except for DCVs (purple), the size of SVs was color-coded (top). The same code also applies to Figure 9 and Movies 2–5. ***C***, Zoomed-in view of the dashed-box area in ***A*** with arrows pointing to putative proteins on the postsynaptic membrane: glutamate receptors, red; other cleft structures, yellow; PSD filaments, blue. ***D***, Scatter plot of length and width dimensions of the particles on the postsynaptic membrane at the synaptic cleft side. Red dots are putative glutamate receptors, and yellow dots are putative nonreceptor structures identified by visual inspection. The sizes of putative receptors (length: 12.1 ± 1.4 nm; width: 8.6 ± 1.4 nm, *n* = 81) are similar to that of extracellular domains of the crystal structures of AMPAR (green; length: 12.0 ± 0.2 nm; width: 10.5 ± 2.4 nm) and NMDAR (magenta; length: 10.5 ± 0.2 nm; width: 10.3 ± 1.4 nm; see detailed calculation of averaged dimensions in Materials and Methods). ***D1***, Averaged 2D image of all particles in the red cluster in ***D***. ***D2***, ***D1*** with AMPAR (green) and NMDAR (magenta) superposed. ***E***, ***F***, Segmented structures on the postsynaptic membrane either superposed on a 1.54-nm-thick (gray) tomographic slice (***E***) or 90°-rotated (***F***) to reveal their deposition on the postsynaptic membrane (cyan). Structures were colored as follows: putative glutamate receptors, red; putative nonreceptor structures on the cleft side, yellow; putative scaffolding proteins on the cytoplasmic side, blue. ***G***, Four types of glutamate receptor-like particles with their interactions on the cytoplasmic side. ***G1***, NMDAR-like structure (extracellular domain: red) had a ∼10 nm globular cytoplasmic domain (pink), which linked to one filamentous structure (blue). ***G2***, ***G3***, AMPAR-like structures (extracellular domain; red) linked to one and two filamentous structures (blue). ***G4***, AMPAR-like structure with no associated filamentous structure. The postsynaptic membrane in all four panels is shown in cyan.

**Figure 9.**
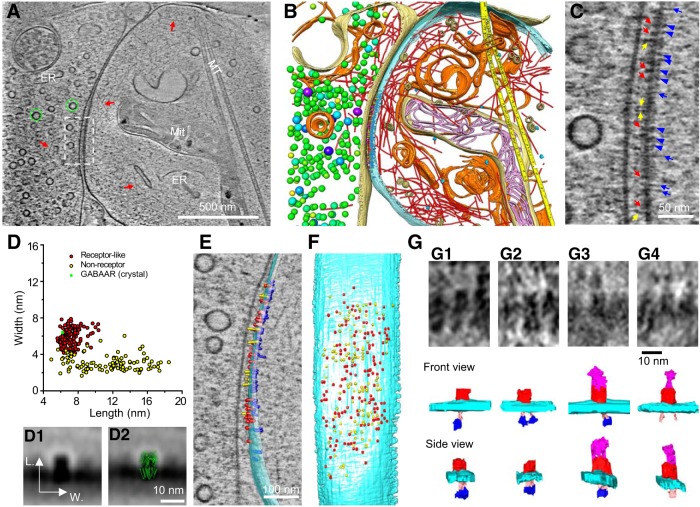
Putative receptors and scaffolding proteins in an inhibitory synapse. ***A***, An 8.7-nm-thick tomographic slice of an inhibitory synapse. Green circles, SVs; red arrows, actin filaments; ER, endoplasmic reticulum; Mit, mitochondria; MT, microtubule. ***B***, 3D segmented structures of the whole tomogram (∼370 nm thickness) of the same synapse shown in ***A*** rendered as surface, colored the same as the labels in Fig. 8*B* except for postsynaptic vesicles (beige). ***C***, Zoomed-in view of the dashed-box area in ***A*** with arrows and arrowheads pointing to particles attached to the postsynaptic membrane. Putative receptors, Red arrows; putative adhesion molecules, yellow arrows; short PSD particles, blue arrowheads; and long PSD particles, blue arrows. ***D***, Scatter plot of length and width dimensions of the structures on the postsynaptic membrane at the synaptic cleft side. Red dots are putative GABA_A_Rs, and yellow dots are putative nonreceptor structures identified by visual inspection. The mean sizes of putative receptors (length: 7.1 ± 0.9 nm; width: 5.9 ± 0.9 nm, *n* = 143) are close to those of the extracellular domain of the crystal structures of GABA_A_Rs (green; length: 6.2 ± 0.1 nm; width: 6.4 ± 0.1 nm; see detailed calculation of averaged dimensions in Materials and Methods). ***D1***, Averaged 2D image of all particles in the red cluster in ***D***. ***D2***, D1 with GABA_A_R (green) superposed. ***E***, ***F***, Segmented structures on the postsynaptic membrane either superposed on a 1.54-nm-thick (gray) tomographic slice (***E***) or 90° rotated (***F***) to reveal their position on the postsynaptic membrane (cyan). Putative GABA_A_R, Red; putative nonreceptor structures in the cleft, yellow; putative scaffolding proteins, blue. ***G***, Typical GABA_A_R-like structures and their interactions at cytoplasmic and cleft side. GABA_A_R-like structures (extracellular domain: red) each linked to one or two hammer-like structures, which had a dense “head” (blue) and a thin “neck” (pink), at cytoplasmic side (***G1–G3***). Some of GABA_A_R-like structures only linked to two “necks” (***G4***). Additionally, some of GABA_A_R-like structures were connected to putative adhesion molecules (magenta) in the extracellular side (***G3***, ***G4***).

We used 10 pregnant rats at gestational day 18 (each had 8–10 fetuses), including seven pregnant rats for direct cryo-EM imaging, and three for cryo-CLEM imaging. Ninety tomograms containing 101 synapses from eight grids were collected using cryo-ET without CLEM. Among them, 49 tomograms (55 synapses) in six grids were collected using a Tecnai F20 equipped with a CCD camera (including all examples in Figs. 1*B*, 2*B–G*, 4*A*,*B*, 5*A*). Later in the study, a Titan Krios equipped with a K2 camera became available and was used to collect 31 tomograms (36 synapses) in one grid (including the example shown in Fig. 2*A*). Additionally, a Titan Krios equipped with K2 camera, VPP, and GIF was used to collect 10 tomograms (10 synapses) in one grid (including the examples in Figs. 6*A*,*B*,*G*, 7–9, Movies 1–5). For cryo-CLEM imaging, only the Tecnai F20 was used to collect 22 tomograms (22 synapses) from four grids (including the examples in Figs. 1*D*, 3*A*,*B*, 5*B*,*C*).

To differentiate excitatory and inhibitory synapses, we collected correlative tomograms containing 14 identified excitatory synapses and eight inhibitory synapses for PSD characterization. An additional 90 synapses with clear PSD from noncorrelative tomograms were pooled together with the identified synapses for cluster analysis of PSD characteristics. For analysis of vesicle shapes, 16,476 vesicles in 35 excitatory synapses, and 4766 vesicles in 15 inhibitory synapses were used for 2D analysis, and 38 selected vesicles in five excitatory synapses and 102 vesicles in five inhibitory synapses were used for 3D analysis. For identifying putative receptors, 145 particles on the postsynaptic membrane of an excitatory synapse and 252 particles on an inhibitory synapse, both obtained with a Titan Krios equipped with K2 camera, VPP, and GIF, were analyzed.

All measurements are presented in the text as mean ± SD. The two-sample Kolmogorov–Smirnov test was used to compare the distributions of the fraction of ellipsoidal vesicles in excitatory versus inhibitory synapse populations.

## Results

### Cryo-ET of synapses in intact primary neurons grown on EM grids

To observe the *in situ* ultrastructure of intact hippocampal synapses, we directly grew hippocampal neurons on gold EM grids, which were plunge-frozen in liquid ethane at 14–18 DIV. This method preserved the structure of vitrified neuronal synapses near their native form, as evidenced by the smooth membranous and cytoskeletal structures (Fig. 1*B2–B4*). Structural deformations commonly seen in conventional EM (Korogod et al., 2015) were not detected in these frozen-hydrated samples.

To find synapses in these samples at low electron dosage (to minimize radiation damage), we usually started from selected grid squares covered by thin ice that contained many neurites (Fig. 1*B2*). We then took a series of single-projection images at high magnification along dendrites to look for synapse-like structures with characteristic features, including closely apposed membranes, with one of which containing a dense population of vesicles of similar sizes, and a relatively uniform cleft in between (Fig. 1*B3*). These membranous structures are easily identifiable under cryo-EM, likely due to higher phase contrast of phospholipids than amorphous background water. In our experiments, only synaptic contacts with approximately normal orientation (i.e., the presynaptic and postsynaptic compartment do not overlap in the single-projection image) were selected for further study, as other contacts could not be easily identified as synapses. These features would become more distinct, along with other fine structural details, in the 3D tomogram reconstructed based on the tilt series collected for each synapse (Fig. 1*B4*). Besides the above characteristic features, the synaptic cleft also contains transcleft filaments and an electron-dense intercleft band similar to the “intermediate band” described previously (Gray, 1959; Fig. 1*B4*, inset).

Based on the above criteria, we identified 101 synapses of various sizes, shapes, and ultrastructural details in 90 tomograms (Fig. 2). Some of the synapses were formed directly on dendritic shafts, with microtubules readily visible in the postsynaptic compartment (Fig. 2*A*). More synapses were formed onto probable spines (Fig. 2*B*,*C*), with a mushroom-like postsynaptic compartment containing no microtubules and a thin neck linking it to the dendrite, as more clearly viewed in 3D tomograms (Fig. 1*B4*). In most synapses, a thick electron density was observed near the postsynaptic membrane (Fig. 2*A*,*B*), analogous to the PSDs of excitatory synapses described in previous studies using conventional EM (Colonnier, 1968; Peters and Palay, 1996). Intriguingly, we also found that ∼18% (18 of 101) of synapses had no “typical” thick PSD structure, but a distinct thin sheet-like structure near the postsynaptic membrane (Fig. 2*C*). This thin sheet-like structure, which has not been reported previously, is reminiscent of the thickened postsynaptic membrane observed in some “symmetric” inhibitory synapses (Colonnier, 1968; Peters and Palay, 1996), as well as the postsynaptic specialization in the glycinergic synapses in the anteroventral cochlear nucleus (Tatsuoka and Reese, 1989). We suspected that such thin sheets were PSDs of inhibitory hippocampal synapses. Indeed, in the few tomograms that captured multiple spines forming synapses onto the same presynaptic bouton or different boutons of the same axon, the seven pairs of spines we observed sharing the same presynaptic cell were always the same type, with either “thick” PSDs (four pairs; Fig. 2*D*) or “thin sheet-like” PSDs (three pairs; Fig. 2*E*). In contrast, when separate boutons formed synapses on the same postsynaptic spine, the corresponding PSDs could be the same or different types (Fig. 2*F*,*G*). This suggests that cryo-ET reveals distinct PSD features of intact excitatory and inhibitory synapses in their native state.

### Identification of excitatory and inhibitory synapses by cryo-CLEM

To unambiguously identify the types of individual synapses visualized by cryo-ET, we developed a cryo-CLEM system (Fig. 1*A*) that took advantage of the specificity of fluorescent protein tagging. In this system, a cryo-chamber built to fit on a light microscope (Fig. 1*C*) can accept an EM cryo-holder through a side port to position the EM grid above the objective lens of the light microscope. This design makes it possible to shuttle the EM cryoholder between light and electron microscopes without repeated sample transfer, thus minimizing ice contamination and grid damage. For fiducial markers, we used the patterned carbon holes on Quantifoil EM grids that can be visualized by both bright-field LM and EM. Based on these patterns, accurate correlation between LM and EM was obtained using a custom-developed program (see Materials and Methods). This approach differs from existing cryo-CLEM methods that rely on the use of large (100–200 nm) fluorescent beads (Schorb and Briggs, 2014; Liu et al., 2015), which may interfere with sample imaging.

We used lentivirus-mediated overexpression of PSD-95-EGFP and mCherry-gephyrin to specifically label glutamatergic and GABAergic synapses, respectively. LM and EM images obtained from different stages of cryo-CLEM are shown in Figure 1*D*. The contrast of the fluorescence images was adjusted for easier visualization of putative synapses seen as fluorescent puncta. Note that the size of a fluorescent punctum does not reflect the true size of a synapse because of limited optical resolution. Using this system, we collected 14 excitatory and eight inhibitory synapses that were identified based on their colocalization with PSD-95-EGFP fluorescence (Fig. 3*A*) and mCherry-gephyrin fluorescence (Fig. 3*B*), respectively. The EM images of these synapses were virtually indistinguishable from those without fluorescent protein labeling, indicating that the overexpression of these tagged scaffolding molecules did not significantly alter synaptic ultrastructure.

Among the 22 synapses identified by fluorescence, and the 101 synapses obtained by cryo-ET only, we observed docked and sometimes partially fused vesicles at the presynaptic membrane (Fig. 2*A*,*B*), but no distinctive high-density “active zone” structure in the presynaptic area (Fig. 2), as described in previous studies using conventional EM (Phillips et al., 2001; Südhof, 2012). By contrast, the postsynaptic sides contained distinctive densities (Figs. 2, 3). Thick (>20 nm) PSD structures next to the plasma membrane were easily identifiable in 13 of the 14 excitatory synapses (Fig. 3*A*) and spanned nearly the entire area of the uniform synaptic cleft (Fig. 3*A1*). The existence of such thick PSDs is consistent with the common belief that excitatory synapses are “asymmetric,” with dense molecular scaffolds on the postsynaptic side (Colonnier, 1968; Peters and Palay, 1996). In contrast, nearly all (seven of eight) inhibitory synapses identified by cryo-CLEM (Fig. 3*B*) had distinct thin sheet-like PSD (thin PSD for short) positioned in parallel and close to the postsynaptic membrane (Fig. 3*B1*). Thus, the previously termed “symmetric” inhibitory synapses are in fact asymmetric under cryo-ET.

### Quantitative analyses of PSD structures in excitatory and inhibitory synapses

By plotting the mean pixel density as a function of its distance to the postsynaptic membrane, we quantified the PSD profiles of the above 20 identified synapses together with 90 additional synapses with visible PSDs from the 101 synapses obtained with cryo-ET but not CLEM (Fig. 4*A*,*B*). The curve contains two major peaks representing the presynaptic and postsynaptic membranes. There are also two smaller peaks on the curve, one between the presynaptic and postsynaptic membrane, representing an electron-dense band within the synaptic cleft reported previously as an intermediate band (Gray, 1959), and the other to the right of the postsynaptic membrane defined as “PSD peak,” presumably indicating a postsynaptic proteinaceous layer. This quantitative approach allowed us to identify *d1*, the peak position of the PSD, and *d2*, which provides a measure of the thickness of the PSD (Fig. 4*A2*,*B2*). Scatter plot of PSD thickness and PSD peak positions of all synapses that contain visible PSDs shows two well-defined clusters, thick and thin, which overlap with the two distinct populations formed by the CLEM-identified excitatory and inhibitory synapses, respectively (Fig. 4*C*). Thus, the distinct PSD patterns detected by cryo-ET can be used as hallmarks to distinguish between the two types of synapses.

With the PSD pattern as an unequivocal criterion, we systematically characterized presynaptic and postsynaptic features in all 110 synapses with visible PSDs analyzed above, including 85 excitatory synapses and 25 inhibitory synapses. Their postsynaptic densities exhibit distinct PSD peak positions (14.7 ± 3.0 nm, *n* = 85 for excitatory synapse; 9.1 ± 1.1 nm, *n* = 25 for inhibitory synapse) and thickness (32.7 ± 7.7 nm, *n* = 85 for excitatory synapse; 12.3 ± 1.8 nm, *n* = 25 for inhibitory synapse). Compared with the uniformly thin PSDs of inhibitory synapses, the thick PSDs of excitatory synapses exhibit substantial variability (Fig. 4*C*,*D*). By averaging all density profiles for excitatory and inhibitory synapses, respectively, we found that the two types of synapses have similar cleft width (∼26 nm; Fig. 4*E*), in contrast to the previous report of narrower cleft for inhibitory synapses (Peters and Palay, 1996). Inside the cleft, distinctive band-like structures are visible in all synapses (Fig. 4*A*,*B*), as reported previously (Gray, 1959; Zuber et al., 2005). We speculate that these structures are protein complexes involved in cell adhesion (Missler et al., 2012). Intriguingly, the density profile around the presynaptic membrane peak is asymmetric; the density values on the cytoplasmic side are slightly higher than that on the cleft side, especially in excitatory synapses. This presumably reflects extra proteins on the cytoplasmic side of the presynaptic membrane, forming a weak version of the active zone commonly observed in conventional EM (Phillips et al., 2001; Südhof, 2012).

In addition to synapses with visible PSD, we also observed 13 structures that met our criteria for synapses but exhibited no visible PSD (Fig. 5*A*). They differ from the more frequently observed nonsynaptic boutons similar to those reported previously (Bourne et al., 2013), because they had distinct uniform synaptic cleft structures that were absent in the latter. These “PSD-free synapses” could be either excitatory or inhibitory as evidenced from the cryo-CLEM data (Fig. 5*B*,*C*). They might reflect a special transient stage, e.g., at an early phase of synaptogenesis or on the way toward elimination (Klemann and Roubos, 2011). The variation in the existence and thickness of PSDs reflects potentially diverse synaptic subtypes and associated signaling and structural mechanisms.

### Quantitative analyses of synaptic vesicles in excitatory and inhibitory synapses

On the presynaptic side, we found that in both excitatory and inhibitory synapses most SVs are spherical (Fig. 6*A–C*), with an average diameter of ∼40 nm (40.9 ± 5.0 nm, 16,476 vesicles in excitatory synapses; 41.3 ± 6.6 nm, 4766 vesicles in inhibitory synapses; Fig. 6*D*). This differs from the classical EM observation that inhibitory vesicles tend to be smaller than excitatory vesicles (Peters and Palay, 1996), but is consistent with findings using HPF-FS (Tatsuoka and Reese, 1989; Korogod et al., 2015), and with measurements from synaptosomes using cryo-EM (Fernández-Busnadiegoet al., 2010). Intriguingly, we also observed some apparently ellipsoidal vesicles in both types of synapses (Fig. 6*A*,*B*). To quantify the shape of different vesicles, we first performed 2D analysis, based on the maximal cross section of each SV in the *x–y* plane, of 19,056 SVs in both excitatory and inhibitory synapses. This analysis identified a relatively small population of ellipsoidal vesicles with major/minor axis ratio significantly >1 (Fig. 6*E*). Interestingly, although ellipsoidal vesicles were found in both excitatory and inhibitory synapses, more were found in the latter (8.5 ± 9%, *n* = 35 in excitatory synapses; 16.9 ± 11%, *n* = 15 in inhibitory synapses; *p* = 0.006, two-sample Kolmogorov–Smirnov test). Indeed, 5 of 15 inhibitory synapses analyzed (in contrast to 3 of 35 excitatory synapses) had ≥20% vesicles that were ellipsoidal (Fig. 6*F*).

The true 3D shape of the vesicles cannot be determined by 2D analysis alone. For example, if the 2D cross section of a vesicle is circular, its 3D shape can be either spherical, “discus-shaped” (i.e., oblate spheroid), or “olive-shaped” (i.e., prolate spheroid; Fig. 6*G*). Therefore, we measured the three principal axes of each vesicle using a 3D fitting program (see Materials and Methods) similar to a method described previously (Kukulski et al., 2012). Measurements of axes with this program on 70 ellipsoidal vesicles and 70 of their neighboring spherical vesicles in five excitatory and five inhibitory synapses (in high-resolution tomograms obtained with VPP, electron filtering, and counting) revealed that most ellipsoidal vesicles were discus-shaped rather than olive-shaped for both excitatory and inhibitory synapses (Fig. 6*H*). The ellipticity of the ellipsoidal vesicles varied widely (Fig. 6*H*), which may reflect their different compositions and functional roles in synaptic transmission.

### Visualization of putative receptors and scaffolding proteins in individual synapses

New tools that enable cryo-ET to achieve higher resolution, including direct electron detection, VPP, and electron energy filter, have facilitated characterization of molecular complexes, such as proteasome in intact cultured neurons (Asano et al., 2015). Using these tools, we obtained high-quality tilt series (Fig. 7*A*,*B*; Movie 1) and tomograms with high contrast and high resolution, permitting visualization of features, such as the two leaflets of the membrane bilayer, microtubule protofilaments, and putative proteasomes (Fig. 7*C–E*). Two high-quality synaptic tomograms obtained using VPP and electron filtering and counting, were selected for further study. One is a spine excitatory synapse with thick PSD structure. The other is a shaft inhibitory synapse with thin sheet-like PSD.

Movie 1. Tilt series of an excitatory synapse. This movie shows the tilt series of an excitatory synapse (same data as in Fig. 8*A*) obtained using VPP, and electron filtering and counting technologies. Structural features, such as SVs, mitochondria, microtubules, and the endoplasmic reticulum in the synapse, can be visualized directly. Black dots of 15 nm diameter are gold beads used as fiducial markers for image alignment. 10.1523/JNEUROSCI.1548-17.2017.video.1

In the excitatory synapse (Fig. 8*A*), large features, such as membranous organelles and ribosomes, as well as actin and microtubule filaments with ultrastructural details, were readily identified and segmented (Fig. 8*B*; Movie 2). Furthermore, numerous particles and filamentous structures of various sizes and shapes were visualized within and across presynaptic and postsynaptic compartments (Fig. 8*C*). These structures were presumably individual protein molecules and complexes. Of special interest were particles near the postsynaptic membrane, some of which have shapes similar to that known for glutamate receptors, including NMDARs and AMPARs, which constitute a major fraction of the postsynaptic membrane proteins (Valtschanoff and Weinberg, 2001; Chen et al., 2008; Dani et al., 2010; Jacob and Weinberg, 2015). We thus visually classified these particles as putative “glutamate receptors,” and defined the remaining particles visible on the cleft side of the postsynaptic membrane as “nonreceptor” particles (Fig. 8*C*). Plotting the length and width of all these particles reveals that the visually identified putative glutamate receptors form a cluster, although not well separated from the nonreceptor particles (Fig. 8*D*). The average length (12.1 ± 1.4 nm, *n* = 81) and width (8.6 ± 1.4 nm, *n* = 81) of particles in this cluster are similar to those of the extracellular domain of AMPARs (length: 12.0 ± 0.2 nm; width: 10.5 ± 2.4 nm) and NMDARs (length: 10.5 ± 0.2 nm; width: 10.3 ± 1.4 nm) based on their crystal structures (see detailed calculation of averaged dimensions in Materials and Methods; Fig. 8*D–D2*). In total, this synapse contained 81 putative glutamate receptors, intermingled with other membrane proteins to occupy the surface of the postsynaptic membrane area (Fig. 8*E*,*F*; Movie 3). This number agrees with estimates of the total number of AMPARs and NMDARs in a glutamatergic synapse based on quantitative immuno-EM (Nusser et al., 1998b; Takumi et al., 1999), quantitative mass spectrometry (Sheng and Hoogenraad, 2007; Lowenthal et al., 2015), and visual identification with ET after HPF-FS (Chen et al., 2008, 2015).

Movie 2. Structures of an excitatory synapse. This movie shows the tomogram of the same synapse as in Figure 8*A*,*B*, displayed as *z*-stack and 3D surface rendering of the segmented structures, including presynaptic (light yellow) and postsynaptic (cyan) membrane, mitochondrial membrane (outer membrane, gold; inner membrane, light pink), endoplasmic reticulum or endosomes (orange), microtubules (yellow), ribosome-like structures (cyan), putative actin filaments (red), presynaptic putative adhesion molecules (magenta), postsynaptic putative adhesion molecules (yellow), glutamate receptor-like particles (red), PSD filaments attached to the postsynaptic membrane (blue), and PSD filaments away from the postsynaptic membrane (purple). Except for dense core vesicles (purple), all other spherical and ellipsoidal shapes are SVs and their varied colors reflect their varying sizes as shown in Figure 8*B*. 10.1523/JNEUROSCI.1548-17.2017.video.2

Among the 81 receptor-like structures in the excitatory synapse, 16 displayed a clear globular density (∼10 nm in diameter) on the cytoplasmic side (Fig. 8*G1*). Such globular densities are unlikely to belong to the MAGUK (membrane-associated guanylate kinase)-family proteins, which have filamentous shapes (Nakagawa et al., 2004; Chen et al., 2008, 2011). We thus suspect that these 16 structures are likely NMDARs, known to have much larger cytoplasmic domains than AMPARs (Chen et al., 2008). Of the remaining 65 receptor-like structures, 11 had relatively low image quality and thus prevented classification based on their cytoplasmic structures, whereas 54 structures could be visually identified as AMPAR-like structures based on the lack of high globular density. Among them, 44 were found to each link to one or two filamentous structure that might represent PSD-95 or similar MAGUK-family proteins (Fig. 8*G2*,*G3*). These putative scaffolding structures appeared to contact the cytoplasmic side of AMPAR-like structures (Fig. 8*G2*,*G3*), reminiscent of PSD-95 anchoring AMPAR through its interaction with stargazin, which binds to the side of the AMPAR (Meyer et al., 2004; Nakagawa et al., 2006). We also observed 10 AMPAR-like structures not associated with any PSD-95-like structures (Fig. 8*G4*). Most of these PSD-95-like structures linking to AMPAR-like structures were in near-perpendicular orientation with respect to the postsynaptic membrane (Fig. 8*G*). Together with ∼200 similar filaments connecting directly to the membrane, they form a set of “vertical pillars” to shape an overall core structure of the PSD (Movie 3), as also seen by ET of samples prepared using HPF-FS (Chen et al., 2008).

Movie 3. Molecular organization of putative membrane proteins in the excitatory synapse. This movie shows the subvolume tomogram (same data as in Fig. 8*E*,*F*) of the synapse as a *z*-stack, and segmentation of its structures, including presynaptic membrane (light yellow), postsynaptic membrane (cyan), presynaptic putative adhesion molecules (magenta), postsynaptic putative adhesion molecules (yellow), putative glutamate receptors (red), PSD filaments attached to the postsynaptic membrane (blue), and away from the postsynaptic membrane (purple). Putative proteins (deep cyan) that link synaptic vesicles (green or cyan) with presynaptic membrane were also rendered. 10.1523/JNEUROSCI.1548-17.2017.video.3

A high-resolution tomogram of an inhibitory synapse also revealed rich ultrastructural details (Fig. 9*A–C*; Movie 4). On the cleft side of the postsynaptic membrane, many particles (Fig. 9*C*) were found with shapes similar to that of the type-A GABA_A_Rs, the primary inhibitory transmitter receptor in these hippocampal neurons (Bi and Poo, 1998; Nusser et al., 1998a). With visual inspection, we provisionally identified ∼143 particles as GABA_A_R, and ∼109 other particles visible on the extracellular side of the postsynaptic membrane as “nonreceptor” particles that likely represent other synaptic proteins, such as adhesion molecules. Plotting the length and width of all these particles revealed that the visually identified putative GABA_A_Rs formed a cluster, and that the sizes of these putative GABA_A_Rs (length: 7.1 ± 0.9 nm; width: 5.9 ± 0.9 nm, *n* = 143) were similar to those of the extracellular domain of GABA_A_R based on its crystal structure (length: 6.2 ± 0.1 nm; width: 6.4 ± 0.1 nm; Fig. 9*D–D2*). Note that the averaged putative GABA_A_R, similar to the averaged putative glutamate receptor, is surrounded by a “halo” (Figs. 8*D1*, 9*D1*), which could be partially due to fringes arising from uncorrected contrast transfer function. However, for individual particles, such effects appeared to be minimal and did not affect the measurements of particle sizes. Most putative nonreceptor particles were uniformly skinny but with variable lengths (Fig. 9*D*). Within this inhibitory synapse, the 143 putative GABA_A_Rs lying amid 109 nonreceptor membrane protein molecules covered the entire ∼0.1 μm^2^ postsynaptic membrane (Fig. 9*E*,*F*; Movie 5). The number and density of GABA_A_R-like particles are consistent with a previous estimate of 30–200 GABA_A_Rs per GABAergic synapse (1250 receptors/μm^2^; Nusser et al., 1997).

Movie 4. Structures of an inhibitory synapse. This movie shows the tomogram of an inhibitory synapse (same data as in Fig. 9*A*,*B*) displayed as *z*-stack and 3D surface rendering of the segmented structures, including presynaptic membrane (light yellow), postsynaptic membrane (cyan), mitochondrial membrane (outer membrane, gold; inner membrane, light pink), endoplasmic reticulum or endosomes (orange), microtubules (yellow), ribosome-like structures (cyan), putative actin filaments (red), presynaptic putative adhesion molecules (magenta), postsynaptic putative adhesion molecules (yellow), GABA_A_R-like particles (red), PSD particles on the postsynaptic membrane (blue), and postsynaptic vesicles (beige). Additionally, except for dense core vesicles (purple), all other spherical and ellipsoidal shapes are SVs and their varied colors reflect their varying sizes as shown in Figure 8*B*. 10.1523/JNEUROSCI.1548-17.2017.video.4

Movie 5. Molecular organization of putative membrane proteins in the inhibitory synapse. This movie shows the subvolume tomogram (same as the data shown in Fig. 9*E*,*F*) of the inhibitory synapse in the *z*-stack and the segmentation of the structures, including presynaptic membrane (light yellow), postsynaptic membrane (cyan), an SV (green) linked to the presynaptic membrane via putative tethering protein (dark cyan), presynaptic putative adhesion molecules (magenta), postsynaptic putative adhesion molecules (yellow), GABA_A_R-like particles (red), and PSD particles on the postsynaptic membrane (blue). 10.1523/JNEUROSCI.1548-17.2017.video.5

Most GABA_A_R-like particles were associated with one or two “hammer-shaped” structures on the cytoplasmic side, each with a dense “head” and a thin “neck” (Fig. 9*G*). The heads of these hammer-shaped structures were consistently located ∼12 nm from the postsynaptic membrane, and the necks bridged the transmembrane domain between the GABA_A_R-like structure and the dense head (Fig. 9*G1–G3*). We speculate that these hammer-shaped structures are protein complexes containing gephyrin molecules, the major postsynaptic scaffolding component of the inhibitory synapse (Tretter et al., 2012). Some of the GABA_A_R-like structures had only thin necks on their cytoplasmic side and thus lacking the head density (Fig. 9*G4*), suggesting that the neck might be the cytosolic domain of GABA_A_Rs. These putative receptor-linked gephyrin-like structures, together with similar particles not linked to receptor-like particles but also lying ∼12 nm from the postsynaptic membrane, appeared to form a cross-linked matrix, which could provide anchoring sites and structural support for GABA_A_Rs, as previously proposed (Tyagarajan and Fritschy, 2014). Interestingly, we also found many GABA_A_R-like structures also linked to densities on the cleft side (Fig. 9*G3*,*G4*). These densities might represent the cell adhesion molecule neurexin, previously reported to bind directly to GABA_A_Rs (Zhang C et al., 2010).

## Discussion

The complex and highly organized molecular machinery inside neuronal synapses provides the structural basis for synaptic transmission and plasticity. In this study, we have developed an approach of cryocorrelative microscopy to distinguish between excitatory and inhibitory synapses in intact neurons in culture and to visualize their 3D structures in their native state. By quantifying ultrastructural features of >100 hippocampal synapses, we have characterized the ultrastructural features across excitatory and inhibitory central synapses. Because the neurons we used were from the embryonic brain and cultured for only a couple weeks, their synapses may not be as mature as those in the adult brain. Nonetheless, such cultured neurons have been shown to exhibit basic physiological properties of synaptic transmission and plasticity similar to those in more intact preparations, such as brain slices. Thus, it is likely that the basic ultrastructural features we observed also reflect synaptic architecture in the brain, at least during its early development.

Our approach allows for unequivocal differentiation of the ultrastructure of excitatory and inhibitory synapses in hippocampal neurons. Our results show that excitatory synapses have distinct thick PSDs, and inhibitory synapses have more uniformly thin PSDs. These corroborate classic findings regarding the ultrastructure of excitatory and inhibitory synapses (Colonnier, 1968; Gray, 1969), while providing an updated description of different PSD types in their native state. Quantitative analysis reveals that excitatory PSDs have a broad distribution of thickness. This is consistent with the idea that the dynamically interacting PSD molecules may be in a mixed gel/liquid phase (Zeng et al., 2016), and suggests the existence of multiple structural configurations, perhaps reflecting different states of their activation (Dosemeci et al., 2001) and plasticity (Bi and Poo, 1998; Montgomery and Madison, 2004). The existence of multiple functional and plasticity states in excitatory synapses could be critical for optimal learning and memory storage in neuronal circuits, as suggested by theoretical studies (Fusi et al., 2005; Fusi and Abbott, 2007). In contrast, inhibitory PSDs are much more uniformly and regularly organized, consistent with the meager evidence for structural plasticity in such synapses. Intriguingly, there is a “gap” area with relatively lower electron density between the postsynaptic membrane and the PSD peak in both excitatory and inhibitory synapses (see the density profiles of Fig. 4*A2*,*B2*). We suspect that much of this gap reflects the relatively lower protein content here, compared with the PSD peak where more extensive interactions between protein molecules may occur.

High-resolution cryo-ET has allowed visualization of the distinct molecular organization underlying the different functional properties of excitatory and inhibitory synapses. In the excitatory synapse, a set of PSD-95-like filamentous structures formed “vertical pillars” immediately underneath the postsynaptic membrane to organize the thick PSD meshwork. These filaments have not been observed in classic studies with conventional EM (Gray, 1959; Colonnier, 1968; Peters and Palay, 1996) or in later studies by ET with brain slices prepared using HPF-FS (Rostaing et al., 2006; Siksou et al., 2007), but is consistent with more recent observation using HPF-FS and ET in cultures (Chen et al., 2008). The discrepancies could be due to differences in image resolution, or could reflect differences between synapses in slices and cultures. Consistent with previous observations (Chen et al., 2008, 2015), we also found that some of the vertical filaments were linked to putative NMDA-type and AMPA-type glutamate receptors. However, the cytoplasmic domains of the putative receptors we observed are much smaller. We speculate that the previously seen large cytoplasmic domains were due to heavy-metal staining.

In the inhibitory synapse, the thin sheet-like PSD is likely to represent putative gephyrin complexes forming a layer of interacting “heads,” which connect to putative GABA_A_Rs and the postsynaptic membrane through filamentous thin “necks.” This is consistent with the hypothesis that gephyrin molecules form a single layer comprising a hexagonal planar lattice that provides docking sites of GABA_A_Rs (Tretter et al., 2012; Heine et al., 2013). Such interactions could provide a stable structural matrix to ensure efficient synaptic transmission.

On the presynaptic side, fine details of synaptic vesicle organization were revealed, including filamentous structures tethering vesicles to the presynaptic membrane, as observed previously in slices using ET with HPF-FS (Siksou et al., 2007), as well as in slices and isolated synaptosomes using cryo-ET (Fernández-Busnadiegoet al., 2010, 2013). Of considerable interest is the presence of discus-shaped ellipsoidal vesicles in both excitatory and inhibitory synapses. “Pleomorphic” vesicles have been observed since early EM studies of the synapse, and were generally considered an indicator of inhibitory synapses (Uchizono, 1965). The nature of such vesicles has been debated as more recent studies indicated that their occurrence was associated with specific conditions of sample processing (Tatsuoka and Reese, 1989; Peters and Palay, 1996; Korogod et al., 2015). Although synapses of cultured hippocampal neurons may have different characteristics compared with mature synapses in brain slices, our results suggest that ellipsoidal vesicles could exist in both excitatory and inhibitory native synapses, but their existence may not be used as a definitive criterion to classify synapse types.

A technological advantage of our approach was the ability to use cryo-CLEM for unambiguous identification of excitatory and inhibitory synapse types. This is potentially extendable to broader applications, especially in light of functional heterogeneity of synapses in neuronal circuits (Dobrunz and Stevens, 1997; Bi and Poo, 2001; Craig and Boudin, 2001; Vogels and Abbott, 2009; Letellier et al., 2016). Compared with immuno-EM labeling, the use of fluorescent protein tagging ensured high label density, while avoiding additional staining steps that can cause significant structural distortions and artifacts. The platform we developed here uses the same EM cryo-holder for shuttling the sample between the light and electron microscopes. Besides being more convenient for reliable correlation between LM and EM, this method also avoids repeated grid transfers, thus protecting the sample from potential damage and contamination. This, together with our method for accurate correlation between LM and EM, greatly improved the efficiency of our approach, and was key to the success of our cryo-CLEM experiments.

Another advantage of our approach is that direct plunge-freezing of neurons cultured on EM grids at low density prevents unwanted disturbance to the synapses, which was unavoidable in the synaptosome preparations previously used for cryo-ET studies (Fernández-Busnadiego et al., 2010; Shi et al., 2014; Perez de Arce et al., 2015). Therefore, the ultrastructure of intact synapses can be preserved near their physiological state. However, plunge-freezing is limited to monolayer cultured neurons and synapses no more than a few hundred nanometers thick. High-pressure freezing and cryo-sectioning could provide an appropriate tool to extend cryo-ET to native circuits in brain tissue (Zuber et al., 2005). By implementing the latest cryo-ET technologies, including VPP, electron energy filter, and direct electron detection, which greatly improve resolution and signal-to-noise ratio (Danev et al., 2014; Fukuda et al., 2015), we were able to visualize synaptic ultrastructural features. Individual molecules in the synapses, such as GABA_A_Rs previously not accessible, could be identified, localized, and counted, thus providing a straightforward way to study key synaptic proteins in individual synapses. With further technical improvements along the lines outlined here, future studies with 3D classification and subtomogram averaging could identify additional synaptic proteins more confidently, and reveal finer structural details of protein complexes *in situ*.

## References

[B1] Asano S, Fukuda Y, Beck F, Aufderheide A, Förster F, Danev R, Baumeister W (2015) Proteasomes. A molecular census of 26S proteasomes in intact neurons. Science 347:439–442. 10.1126/science.1261197 25613890

[B2] Bi GQ, Poo MM (1998) Synaptic modifications in cultured hippocampal neurons: dependence on spike timing, synaptic strength, and postsynaptic cell type. J Neurosci 18:10464–10472. 9852584 10.1523/JNEUROSCI.18-24-10464.1998 PMC6793365

[B3] Bi G, Poo M (2001) Synaptic modification by correlated activity: Hebb's postulate revisited. Annu Rev Neurosci 24:139–166. 10.1146/annurev.neuro.24.1.139 11283308

[B4] Bourne JN, Chirillo MA, Harris KM (2013) Presynaptic ultrastructural plasticity along CA3→CA1 axons during long-term potentiation in mature hippocampus. J Comp Neurol 521:3898–3912. 10.1002/cne.23384 23784793 PMC3838200

[B5] Brandt F, Carlson LA, Hartl FU, Baumeister W, Grünewald K (2010) The three-dimensional organization of polyribosomes in intact human cells. Mol Cell 39:560–569. 10.1016/j.molcel.2010.08.003 20797628

[B6] Burette AC, Lesperance T, Crum J, Martone M, Volkmann N, Ellisman MH, Weinberg RJ (2012) Electron tomographic analysis of synaptic ultrastructure. J Comp Neurol 520:2697–2711. 10.1002/cne.23067 22684938 PMC3856703

[B7] Chen X, Winters C, Azzam R, Li X, Galbraith JA, Leapman RD, Reese TS (2008) Organization of the core structure of the postsynaptic density. Proc Natl Acad Sci U S A 105:4453–4458. 10.1073/pnas.0800897105 18326622 PMC2393784

[B8] Chen X, Nelson CD, Li X, Winters CA, Azzam R, Sousa AA, Leapman RD, Gainer H, Sheng M, Reese TS (2011) PSD-95 is required to sustain the molecular organization of the postsynaptic density. J Neurosci 31:6329–6338. 10.1523/JNEUROSCI.5968-10.2011 21525273 PMC3099547

[B9] Chen X, Levy JM, Hou A, Winters C, Azzam R, Sousa AA, Leapman RD, Nicoll RA, Reese TS (2015) PSD-95 family MAGUKs are essential for anchoring AMPA and NMDA receptor complexes at the postsynaptic density. Proc Natl Acad Sci U S A 112:E6983–E6992. 10.1073/pnas.1517045112 26604311 PMC4687590

[B10] Colonnier M (1968) Synaptic patterns on different cell types in the different laminae of the cat visual cortex. An electron microscope study. Brain Res 9:268–287. 10.1016/0006-8993(68)90234-5 4175993

[B11] Craig AM, Boudin H (2001) Molecular heterogeneity of central synapses: afferent and target regulation. Nat Neurosci 4:569–578. 10.1038/88388 11369937

[B12] Danev R, Buijsse B, Khoshouei M, Plitzko JM, Baumeister W (2014) Volta potential phase plate for in-focus phase contrast transmission electron microscopy. Proc Natl Acad Sci U S A 111:15635–15640. 10.1073/pnas.1418377111 25331897 PMC4226124

[B13] Dani A, Huang B, Bergan J, Dulac C, Zhuang X (2010) Superresolution imaging of chemical synapses in the brain. Neuron 68:843–856. 10.1016/j.neuron.2010.11.021 21144999 PMC3057101

[B14] Dobie FA, Craig AM (2011) Inhibitory synapse dynamics: coordinated presynaptic and postsynaptic mobility and the major contribution of recycled vesicles to new synapse formation. J Neurosci 31:10481–10493. 10.1523/JNEUROSCI.6023-10.2011 21775594 PMC6622636

[B15] Dobrunz LE, Stevens CF (1997) Heterogeneity of release probability, facilitation, and depletion at central synapses. Neuron 18:995–1008. 10.1016/S0896-6273(00)80338-4 9208866

[B16] Dosemeci A, Tao-Cheng JH, Vinade L, Winters CA, Pozzo-Miller L, Reese TS (2001) Glutamate-induced transient modification of the postsynaptic density. Proc Natl Acad Sci U S A 98:10428–10432. 10.1073/pnas.181336998 11517322 PMC56977

[B17] Dürr KL, Chen L, Stein RA, De Zorzi R, Folea IM, Walz T, Mchaourab HS, Gouaux E (2014) Structure and dynamics of AMPA receptor GluA2 in resting, pre-open, and desensitized states. Cell 158:778–792. 10.1016/j.cell.2014.07.023 25109876 PMC4263325

[B18] Eccles JC (1964) The physiology of synapses. Berlin, Germany: Springer.

[B19] Fernández-Busnadiego R, Zuber B, Maurer UE, Cyrklaff M, Baumeister W, Lucic V (2010) Quantitative analysis of the native presynaptic cytomatrix by cryo-electron tomography. J Cell Biol 188:145–156. 10.1083/jcb.200908082 20065095 PMC2812849

[B20] Fernández-Busnadiego R, Asano S, Oprisoreanu AM, Sakata E, Doengi M, Kochovski Z, Zürner M, Stein V, Schoch S, Baumeister W, Lucić V (2013) Cryo-electron tomography reveals a critical role of RIM1alpha in synaptic vesicle tethering. The Journal of cell biology 201:725–740. 10.1083/jcb.201206063 23712261 PMC3664715

[B21] Fitzgibbon A, Pilu M, Fisher RB (1999) Direct least square fitting of ellipses. IEEE Trans Pattern Anal Mach Intell 21:476–480. 10.1109/34.765658

[B22] Fukuda Y, Laugks U, Lučić V, Baumeister W, Danev R (2015) Electron cryotomography of vitrified cells with a Volta phase plate. J Struct Biol 190:143–154. 10.1016/j.jsb.2015.03.004 25770733

[B23] Fusi S, Abbott LF (2007) Limits on the memory storage capacity of bounded synapses. Nat Neurosci 10:485–493. 10.1038/nn1859 17351638

[B24] Fusi S, Drew PJ, Abbott LF (2005) Cascade models of synaptically stored memories. Neuron 45:599–611. 10.1016/j.neuron.2005.02.001 15721245

[B25] Gray EG (1959) Axo-somatic and axo-dendritic synapses of the cerebral cortex: an electron microscope study. J Anat 93:420–433. 13829103 PMC1244535

[B26] Gray EG (1969) Electron microscopy of excitatory and inhibitory synapses: a brief review. Prog Brain Res 31:141–155. 10.1016/S0079-6123(08)63235-5 4899407

[B27] Harlow M, Ress D, Koster A, Marshall RM, Schwarz M, McMahan UJ (1998) Dissection of active zones at the neuromuscular junction by EM tomography. J Physiol Paris 92:75–78. 10.1016/S0928-4257(98)80141-1 9782447

[B28] Harris KM, Weinberg RJ (2012) Ultrastructure of synapses in the mammalian brain. Cold Spring Harb Perspect Biol 4:pii:a005587. 10.1101/cshperspect.a005587 22357909 PMC3331701

[B29] Heine M, Karpova A, Gundelfinger ED (2013) Counting gephyrins, one at a time: a nanoscale view on the inhibitory postsynapse. Neuron 79:213–216. 10.1016/j.neuron.2013.07.004 23889929

[B30] Hrabe T, Chen Y, Pfeffer S, Cuellar LK, Mangold AV, Förster F (2012) PyTom: a python-based toolbox for localization of macromolecules in cryo-electron tomograms and subtomogram analysis. J Struct Biol 178:177–188. 10.1016/j.jsb.2011.12.003 22193517

[B31] Hurbain I, Sachse M (2011) The future is cold: cryo-preparation methods for transmission electron microscopy of cells. Biol Cell 103:405–420. 10.1042/BC20110015 21812762

[B32] Imig C, Min SW, Krinner S, Arancillo M, Rosenmund C, Südhof TC, Rhee J, Brose N, Cooper BH (2014) The morphological and molecular nature of synaptic vesicle priming at presynaptic active zones. Neuron 84:416–431. 10.1016/j.neuron.2014.10.009 25374362

[B33] Jacob AL, Weinberg RJ (2015) The organization of AMPA receptor subunits at the postsynaptic membrane. Hippocampus 25:798–812. 10.1002/hipo.22404 25524891 PMC4472633

[B34] Jung JH, Szule JA, Marshall RM, McMahan UJ (2016) Variable priming of a docked synaptic vesicle. Proc Natl Acad Sci U S A 113:E1098–E1107. 10.1073/pnas.1523054113 26858418 PMC4776491

[B35] Klemann CJ, Roubos EW (2011) The gray area between synapse structure and function—Gray's synapse types I and II revisited. Synapse 65:1222–1230. 10.1002/syn.20962 21656572

[B36] Korogod N, Petersen CC, Knott GW (2015) Ultrastructural analysis of adult mouse neocortex comparing aldehyde perfusion with cryo fixation. eLife 4. 10.7554/eLife.05793 26259873 PMC4530226

[B37] Kremer JR, Mastronarde DN, McIntosh JR (1996) Computer visualization of three-dimensional image data using IMOD. J Struct Biol 116:71–76. 10.1006/jsbi.1996.0013 8742726

[B38] Kukulski W, Schorb M, Kaksonen M, Briggs JA (2012) Plasma membrane reshaping during endocytosis is revealed by time-resolved electron tomography. Cell 150:508–520. 10.1016/j.cell.2012.05.046 22863005

[B39] Lee CH, Lü W, Michel JC, Goehring A, Du J, Song X, Gouaux E (2014) NMDA receptor structures reveal subunit arrangement and pore architecture. Nature 511:191–197. 10.1038/nature13548 25008524 PMC4263351

[B40] Letellier M, Park YK, Chater TE, Chipman PH, Gautam SG, Oshima-Takago T, Goda Y (2016) Astrocytes regulate heterogeneity of presynaptic strengths in hippocampal networks. Proc Natl Acad Sci U S A 113:E2685–E2694. 10.1073/pnas.1523717113 27118849 PMC4868440

[B41] Li X, Mooney P, Zheng S, Booth CR, Braunfeld MB, Gubbens S, Agard DA, Cheng Y (2013) Electron counting and beam-induced motion correction enable near-atomic-resolution single-particle cryo-EM. Nat Methods 10:584–590. 10.1038/nmeth.2472 23644547 PMC3684049

[B42] Liu B, Xue Y, Zhao W, Chen Y, Fan C, Gu L, Zhang Y, Zhang X, Sun L, Huang X, Ding W, Sun F, Ji W, Xu T (2015) Three-dimensional super-resolution protein localization correlated with vitrified cellular context. Sci Rep 5:13017. 10.1038/srep13017 26462878 PMC4604464

[B43] Lowenthal MS, Markey SP, Dosemeci A (2015) Quantitative mass spectrometry measurements reveal stoichiometry of principal postsynaptic density proteins. J Proteome Res 14:2528–2538. 10.1021/acs.jproteome.5b00109 25874902 PMC5597335

[B44] Lucić V, Kossel AH, Yang T, Bonhoeffer T, Baumeister W, Sartori A (2007) Multiscale imaging of neurons grown in culture: from light microscopy to cryo-electron tomography. J Struct Biol 160:146–156. 10.1016/j.jsb.2007.08.014 17905597

[B45] Mastronarde DN (2005) Automated electron microscope tomography using robust prediction of specimen movements. J Struct Biol 152:36–51. 10.1016/j.jsb.2005.07.007 16182563

[B46] Mayford M, Siegelbaum SA, Kandel ER (2012) Synapses and memory storage. Cold Spring Harb Perspect Biol 4:pii:a005751. 10.1101/cshperspect.a005751 22496389 PMC3367555

[B47] Megías M, Emri Z, Freund TF, Gulyás AI (2001) Total number and distribution of inhibitory and excitatory synapses on hippocampal CA1 pyramidal cells. Neuroscience 102:527–540. 10.1016/S0306-4522(00)00496-6 11226691

[B48] Meyer G, Varoqueaux F, Neeb A, Oschlies M, Brose N (2004) The complexity of PDZ domain-mediated interactions at glutamatergic synapses: a case study on neuroligin. Neuropharmacology 47:724–733. 10.1016/j.neuropharm.2004.06.023 15458844

[B49] Miller PS, Aricescu AR (2014) Crystal structure of a human GABA_A_ receptor. Nature 512:270–275. 10.1038/nature13293 24909990 PMC4167603

[B50] Milner B, Squire LR, Kandel ER (1998) Cognitive neuroscience and the study of memory. Neuron 20:445–468. 10.1016/S0896-6273(00)80987-3 9539121

[B51] Missler M, Südhof TC, Biederer T (2012) Synaptic cell adhesion. Cold Spring Harb Perspect Biol 4:a005694. 10.1101/cshperspect.a005694 22278667 PMC3312681

[B52] Montgomery JM, Madison DV (2004) Discrete synaptic states define a major mechanism of synapse plasticity. Trends Neurosci 27:744–750. 10.1016/j.tins.2004.10.006 15541515

[B53] Nakagawa T, Futai K, Lashuel HA, Lo I, Okamoto K, Walz T, Hayashi Y, Sheng M (2004) Quaternary structure, protein dynamics, and synaptic function of SAP97 controlled by L27 domain interactions. Neuron 44:453–467. 10.1016/j.neuron.2004.10.012 15504326

[B54] Nakagawa T, Cheng Y, Sheng M, Walz T (2006) Three-dimensional structure of an AMPA receptor without associated stargazin/TARP proteins. Biol Chem 387:179–187. 16497150 10.1515/BC.2006.024

[B55] Nusser Z, Cull-Candy S, Farrant M (1997) Differences in synaptic GABA(A) receptor number underlie variation in GABA mini amplitude. Neuron 19:697–709. 10.1016/S0896-6273(00)80382-7 9331359

[B56] Nusser Z, Hájos N, Somogyi P, Mody I (1998a) Increased number of synaptic GABA(A) receptors underlies potentiation at hippocampal inhibitory synapses. Nature 395:172–177. 10.1038/25999 9744275

[B57] Nusser Z, Lujan R, Laube G, Roberts JD, Molnar E, Somogyi P (1998b) Cell type and pathway dependence of synaptic AMPA receptor number and variability in the hippocampus. Neuron 21:545–559. 10.1016/S0896-6273(00)80565-6 9768841

[B58] Perez de Arce K, Schrod N, Metzbower SW, Allgeyer E, Kong GK, Tang AH, Krupp AJ, Stein V, Liu X, Bewersdorf J, Blanpied TA, Lucić V, Biederer T (2015) Topographic mapping of the synaptic cleft into adhesive nanodomains. Neuron 88:1165–1172. 10.1016/j.neuron.2015.11.011 26687224 PMC4687029

[B59] Peters A, Palay SL (1996) The morphology of synapses. J Neurocytol 25:687–700. 10.1007/BF02284835 9023718

[B60] Pettersen EF, Goddard TD, Huang CC, Couch GS, Greenblatt DM, Meng EC, Ferrin TE (2004) UCSF chimera—a visualization system for exploratory research and analysis. J Comput Chem 25:1605–1612. 10.1002/jcc.20084 15264254

[B61] Phillips GR, Huang JK, Wang Y, Tanaka H, Shapiro L, Zhang W, Shan WS, Arndt K, Frank M, Gordon RE, Gawinowicz MA, Zhao Y, Colman DR (2001) The presynaptic particle web: ultrastructure, composition, dissolution, and reconstitution. Neuron 32:63–77. 10.1016/S0896-6273(01)00450-0 11604139

[B62] Ress DB, Harlow ML, Marshall RM, McMahan UJ (2004) Methods for generating high-resolution structural models from electron microscope tomography data. Structure 12:1763–1774. 10.1016/j.str.2004.07.022 15458626 PMC4312110

[B63] Rostaing P, Real E, Siksou L, Lechaire JP, Boudier T, Boeckers TM, Gertler F, Gundelfinger ED, Triller A, Marty S (2006) Analysis of synaptic ultrastructure without fixative using high-pressure freezing and tomography. Eur J Neurosci 24:3463–3474. 10.1111/j.1460-9568.2006.05234.x 17229095

[B64] Sassoè-Pognetto M, Frola E, Pregno G, Briatore F, Patrizi A (2011) Understanding the molecular diversity of GABAergic synapses. Front Cell Neurosci 5:4. 10.3389/fncel.2011.00004 21713106 PMC3112311

[B65] Schikorski T, Stevens CF (1997) Quantitative ultrastructural analysis of hippocampal excitatory synapses. J Neurosci 17:5858–5867. 9221783 10.1523/JNEUROSCI.17-15-05858.1997 PMC6573206

[B66] Schikorski T, Stevens CF (2001) Morphological correlates of functionally defined synaptic vesicle populations. Nat Neurosci 4:391–395. 10.1038/86042 11276229

[B67] Schorb M, Briggs JA (2014) Correlated cryo-fluorescence and cryo-electron microscopy with high spatial precision and improved sensitivity. Ultramicroscopy 143:24–32. 10.1016/j.ultramic.2013.10.015 24275379 PMC5472196

[B68] Sheng M, Hoogenraad CC (2007) The postsynaptic architecture of excitatory synapses: a more quantitative view. Annu Rev Biochem 76:823–847. 10.1146/annurev.biochem.76.060805.160029 17243894

[B69] Shi L, Du X, Zhou H, Tao C, Liu Y, Meng F, Wu G, Xiong Y, Xia C, Wang Y, Bi G, Zhou JN (2014) Cumulative effects of the ApoE genotype and gender on the synaptic proteome and oxidative stress in the mouse brain. Int J Neuropsychopharmacol 17:1863–1879. 10.1017/S1461145714000601 24810422

[B70] Siksou L, Rostaing P, Lechaire JP, Boudier T, Ohtsuka T, Fejtová A, Kao HT, Greengard P, Gundelfinger ED, Triller A, Marty S (2007) Three-dimensional architecture of presynaptic terminal cytomatrix. J Neurosci 27:6868–6877. 10.1523/JNEUROSCI.1773-07.2007 17596435 PMC6672225

[B71] Sorra KE, Harris KM (2000) Overview on the structure, composition, function, development, and plasticity of hippocampal dendritic spines. Hippocampus 10:501–511. 10.1002/1098-1063(2000)10:5<501::AID-HIPO1>3.0.CO;2-T 11075821

[B72] Südhof TC (2012) The presynaptic active zone. Neuron 75:11–25. 10.1016/j.neuron.2012.06.012 22794257 PMC3743085

[B73] Südhof TC, Malenka RC (2008) Understanding synapses: past, Present, and Future. Neuron 60:469–476. 10.1016/j.neuron.2008.10.011 18995821 PMC3243741

[B74] Suloway C, Pulokas J, Fellmann D, Cheng A, Guerra F, Quispe J, Stagg S, Potter CS, Carragher B (2005) Automated molecular microscopy: the new leginon system. J Struct Biol 151:41–60. 10.1016/j.jsb.2005.03.010 15890530

[B75] Takumi Y, Ramírez-León V, Laake P, Rinvik E, Ottersen OP (1999) Different modes of expression of AMPA and NMDA receptors in hippocampal synapses. Nat Neurosci 2:618–624. 10.1038/10172 10409387

[B76] Tao C, Xia C, Chen X, Zhou ZH, Bi G (2012) Ultrastructural analysis of neuronal synapses using state-of-the-art nano-imaging techniques. Neurosci Bull 28:321–332. 10.1007/s12264-012-1249-z 22833032 PMC5561890

[B77] Tatsuoka H, Reese TS (1989) New structural features of synapses in the anteroventral cochlear nucleus prepared by direct freezing and freeze-substitution. J Comp Neurol 290:343–357. 10.1002/cne.902900304 2592616

[B78] Tretter V, Mukherjee J, Maric HM, Schindelin H, Sieghart W, Moss SJ (2012) Gephyrin, the enigmatic organizer at GABAergic synapses. Front Cell Neurosci 6:23. 10.3389/fncel.2012.00023 22615685 PMC3351755

[B79] Tyagarajan SK, Fritschy JM (2014) Gephyrin: a master regulator of neuronal function? Nat Rev Neurosci 15:141–156. 10.1038/nrn3670 24552784

[B80] Uchizono K (1965) Characteristics of excitatory and inhibitory synapses in the central nervous system of the cat. Nature 207:642–643. 10.1038/207642a0 5883646

[B81] Valtschanoff JG, Weinberg RJ (2001) Laminar organization of the NMDA receptor complex within the postsynaptic density. J Neurosci 21:1211–1217. 11160391 10.1523/JNEUROSCI.21-04-01211.2001 PMC6762240

[B82] Verbeeck J, Van Dyck D, Van Tendeloo G (2004) Energy-filtered transmission electron microscopy: an overview. Spectrochim Acta B 59:1529–1534. 10.1016/j.sab.2004.03.020

[B83] Vogels TP, Abbott LF (2009) Gating multiple signals through detailed balance of excitation and inhibition in spiking networks. Nat Neurosci 12:483–491. 10.1038/nn.2276 19305402 PMC2693069

[B84] Watanabe S, Rost BR, Camacho-Pérez M, Davis MW, Söhl-Kielczynski B, Rosenmund C, Jorgensen EM (2013) Ultrafast endocytosis at mouse hippocampal synapses. Nature 504:242–247. 10.1038/nature12809 24305055 PMC3957339

[B85] Wilhelm BG, Mandad S, Truckenbrodt S, Kröhnert K, Schäfer C, Rammner B, Koo SJ, Claßen GA, Krauss M, Haucke V, Urlaub H, Rizzoli SO (2014) Composition of isolated synaptic boutons reveals the amounts of vesicle trafficking proteins. Science 344:1023–1028. 10.1126/science.1252884 24876496

[B86] Zeng M, Shang Y, Araki Y, Guo T, Huganir RL, Zhang M (2016) Phase transition in postsynaptic densities underlies formation of synaptic complexes and synaptic plasticity. Cell 166:1163–1175.e12. 10.1016/j.cell.2016.07.008 27565345 PMC5564291

[B87] Zhang C, Atasoy D, Araç D, Yang X, Fucillo MV, Robison AJ, Ko J, Brunger AT, Südhof TC (2010) Neurexins physically and functionally interact with GABA(A) receptors. Neuron 66:403–416. 10.1016/j.neuron.2010.04.008 20471353 PMC3243752

[B88] Zhang F, Gradinaru V, Adamantidis AR, Durand R, Airan RD, de Lecea L, Deisseroth K (2010) Optogenetic interrogation of neural circuits: technology for probing mammalian brain structures. Nat Protoc 5:439–456. 10.1038/nprot.2009.226 20203662 PMC4503465

[B89] Zuber B, Nikonenko I, Klauser P, Muller D, Dubochet J (2005) The mammalian central nervous synaptic cleft contains a high density of periodically organized complexes. Proc Natl Acad Sci U S A 102:19192–19197. 10.1073/pnas.0509527102 16354833 PMC1323199

